# MINDflex Training for Cognitive Flexibility in Chronic Pain: A Randomized, Controlled Cross-Over Trial

**DOI:** 10.3389/fpsyg.2020.604832

**Published:** 2020-12-21

**Authors:** Henrik B. Jacobsen, Ole Klungsøyr, Nils I. Landrø, Tore C. Stiles, Bryan T. Roche

**Affiliations:** ^1^Department of Pain Management and Research, Division of Emergencies and Critical Care, Oslo University Hospital, Oslo, Norway; ^2^The Mind-Body Lab, Department of Psychology, Faculty of Social Sciences, University of Oslo, Oslo, Norway; ^3^Oslo Centre for Biostatistics and Epidemiology, Section for Treatment Research, Department of Research and Innovation, Division of Mental Health and Addiction, Oslo University Hospital, Oslo, Norway; ^4^Clinical Neuroscience Research Group, Department of Psychology, Faculty of Social Sciences, University of Oslo, Oslo, Norway; ^5^Department of Psychology, Faculty of Social and Educational Sciences, Norwegian University of Science and Technology, Trondheim, Norway; ^6^Department of Psychology, Maynooth University, Maynooth, Ireland

**Keywords:** pain, Relational Frame Theory (RFT), cognitive training, far transfer, randomized controlled (clinical) trial

## Abstract

Impairments in executive functioning are prevalent in chronic pain conditions, with cognitive inflexibility being the most frequently reported. The current randomized, cross-over trial, piloted a computerized cognitive training (CCT) program based on Relational Frame Theory, targeting improvement in cognitive flexibility. At baseline, 73 chronic pain patients completed testing on pre-selected outcomes of executive functioning, alongside IQ measures. When tested three times over the course of 5 months, there was a drop-out rate of 40% at the third time point, leaving 44 patients who had data at all time points. The results showed that there was a substantial learning effect from the MINDFLEX training and a substantial time-dependent improvement on the primary outcomes of increased flexibility, but that this could not be tied to active training. In conclusion, this small study indicated a learning effect as well as improvement on primary outcomes. Based on the current results, a larger trial with improved feasibility of training is warranted.

## Introduction

It was recently suggested that a generalized cognitive inflexibility impacts the way chronic pain patients attend to, interpret and recollect information; processes often referred to collectively as executive functioning (Van Ryckeghem et al., 2019). Executive functions (EF) is an umbrella term that describes mental processes regulating our behavior, especially in non-routine situations (Diamond, 2013; Goldstein and Naglieri, 2014; Friedman and Miyake, 2017). It has been theorized that the three overlapping, core functions of EF are inhibition, working memory updating, and cognitive flexibility (Miyake and Friedman, 2012; Diamond, 2013).

The general cognitive inflexibility described in chronic pain could be a product of threat monitoring and hypervigilance toward painful stimuli, which initially is adaptive when experiencing pain (Vlaeyen and Linton, 2012). Over time, however, the patient learns through aversive conditioning events (e.g., pain experiences associated directly with specific movements), and a more recently identified process known as derived relational responding (Barnes-Holmes and Roche, 2001; Van Ryckeghem et al., 2019), that they should avoid stimuli even without direct aversive conditioning. Aversive conditioning and derived relational responding thus combine to facilitate the generalization of pain avoidance behaviors along conceptual continua such as verbal categories (see Bennett M. P. et al., 2015; Assaz et al., 2018). This could be described as a process where mental rules or memory representations start guiding behavior *pre-hoc* (e.g., I will not bend my back for any reason or in any context). Such rules develop and generalize through different forms of derived relational responding, creating overarching or generalized operant classes of arbitrarily applicable relational responding where the most well-known is stimulus equivalency (Barnes, 1994).

A *symbolic generalization* of pain avoidance from behavior into verbal behavior is associated with the development of rigid behavior patterns, referred to as a process of cognitive fusion within Acceptance and Commitment Theory (Assaz et al., 2018). Cognitive fusion can be defined as behavior being overly regulated and influenced by thoughts and perceptions, meaning that cognitive events come to dominate behavior over other sources of behavioral regulation such as external contextual cues (Gillanders et al., 2014). The behavior of a cognitively fused patient is shielded from influence by direct contingencies, and the individual will exhibit reduced attention to current contingencies or contextual cues (Baruch et al., 2007; Gillanders et al., 2014; Dymond et al., 2015). Cognitive fusion has been suggested to mediate the relationship between distress and cognitive flexibility making it a target for intervention when attempting to increase cognitive flexibility (Palm and Follette, 2011).

When their executive functioning is tested, chronic pain patients show both cognitive inflexibility (Berryman et al., 2013) and attention biases toward painful stimuli (Higgins et al., 2018; Mazza et al., 2018). These results point to an overall impairment in executive functioning in chronic pain (Miyake and Friedman, 2012). However, it is not known if these deficits are the result of cognitive inflexibility alone (Gillanders et al., 2014), or if this is a more complex interaction between different components of executive function where the lack of flexibility is a bi-product.

The Unity and Diversity model of executive functions argues that there is both considerable overlap and distinct differences between the three core functions of EF (Miyake and Friedman, 2012). If flexibility is either an underlying process or a main reason for a reduced executive functioning in chronic pain, it might be expected that a computerized cognitive training (CCT; Van Ryckeghem et al., 2019) intervention designed to improve cognitive flexibility (i.e., reduce fusion) may lead to improvements in executive function.

Only two studies have rigorously evaluated CCT in different types of chronic pain (Baker et al., 2018; Santos et al., 2018). Neither explicitly targeted cognitive inflexibility, but attempted to improve overall cognitive functioning through training all three core executive functions. Following a 5-week comprehensive intervention in patients with chronic back pain, Baker et al. (2018) demonstrated significant training effects for a global cognitive composite measure (netES = 0.43) and for an executive composite measure (netES = 0.55), but not for attention or reaction time (Baker et al., 2018). Combining transcranial direct stimulation with CCT showed no differences in delta values between active and control groups (Santos et al., 2018). In addition, another study found that in a sample including chronic pain patients, the CCT improved performance in inhibitory control, but not the other executive functions (Aasvik et al., 2017).

To summarize, CCT seems to improve some executive functions, but participants’ progress appear highly context or task dependent (Aasvik et al., 2017; Santos et al., 2018). This indicates so-called near, rather than far-transfer of effects; i.e., effects transfer only to skills of the same type as those trained (Taatgen, 2013). In an attempt to achieve far-transfer, we developed a novel CCT based on principles from Relational Frame Theory [RFT; Hayes, (Barnes-Holmes and Roche, 2001)]. While a full account of RFT is beyond the scope of this introduction, the core claims of RFT are relatively simple: Humans are capable of deriving arbitrary relations among stimuli without direct training (e.g., syllogistic reasoning), and the derivation of stimulus relations is both amenable to learning and its fluency can be enhanced (Gross and Fox, 2009). Importantly, the stimuli in a derived relation transform the functions of the other relation members [e.g., words that participate in derived equivalence relations with conditioned threat words, elicit *derived* threat themselves (Bennett M. et al., 2015; Bennett M. P. et al., 2015; Boyle et al., 2016)].

In the current study, we aimed to evaluate and prototype a CCT based on an emerging behavior-analytic method known as the Function Acquisition Speed Test (FAST) (O’Reilly et al., 2013; Cartwright et al., 2017; Cummins et al., 2018). The FAST assesses the differential rate at which relations between classes of stimuli (words or images) are acquired in two differing training configurations. Specifically, participants are trained to produce a common motor response (usually positional on a computer keyboard) upon the presentation of examples from one of four categories (e.g., pain words, relaxation words, positive words, and negative words). Examples of two categories share a response function (e.g., press a left-hand key), and exemplars of the two remaining categories share another (e.g., press a right-hand key). This configuration is juxtaposed in another block of training for the purpose of assessing which arrangement produces the steepest learning curves (i.e., increase in speed and accuracy of responses) across two finite blocks of training trials. The size and direction of the learning rate differed across training arrangements (blocks), which indicates which pairs of verbal categories are most highly related in the verbal history of the test taker.

While the FAST is usually employed as an assessment of verbal relation strength (i.e., degree of cognitive fusion), we here hypothesize that it can be used as a training method to weaken relations between relations (i.e., decrease fusion) because it operates by essentially attempting to both reinforce and “break” equivalence relations among verbal categories across two training blocks. Upon repeated presentations of the test, the verbal relations employed become more flexible, such that classes can be inter-related in various ways at an increasingly faster rate. This approach is consistent with an Acceptance and Commitment Therapy approach (Hayes et al., 2011), and in particular represents the computer-based delivery of a cognitive defusion exercise (Assaz et al., 2018). We will refer here to the use of the FAST as a CCT for cognitive flexibility as the MINDflex method.

In this study, we employed repeated MINDflex training as a novel CCT for achieving cognitive defusion, or increasing cognitive flexibility, in relation to salient and ideographically selected pain-related stimuli for pain patients. Ideographic stimuli representative of pain, neutral stimuli, positive stimuli and negative stimuli were employed in sequential MINDflex training across several sessions, with the aim of reducing learning rate differentials across the two FAST blocks in each test administration (i.e., a reducing index of cognitive fusion). This method was evaluated using a randomized controlled crossover design.

First, we aimed to show that the MINDflex training was effective in increasing relational flexibility in relation to ideographic salient pain stimuli. Secondly, we aimed to evaluate whether this training was effective in improving cognitive flexibility measured in terms of scores on objective neuropsychological testing using the Cambridge Neuropsychological Test Automated Battery (CANTAB). Thirdly, we aimed to assess whether the amount of training delivered to each patient was related to the degree of impact on patient’s scores on selected CANTAB outcomes.

We hypothesized that the MINDflex training had the capability to improve a composite measure of correct and rapid responding (i.e., fluency) at the MINDflex tasks, exponentially both across multiple brief exposures within sessions and over time (i.e., a reduction in cognitive fusion/an increase in cognitive flexibility; H_1_). We further hypothesized that only participants who had received MINDflex training would improve on neuropsychological tests of cognitive flexibility, measurable immediately after the training period (H_2_), and at 2-month follow-up (H_3_).

## Materials and Methods

### Study Setting

From July 2016 until March 2018 the research group recruited potential candidates from the patient population at the Department of Pain Management and Research, Oslo University Hospital, a tertiary multidisciplinary pain clinic. In addition, information leaflets were circulated to all relevant pain clinics and patient organizations asking general practitioners to refer patients to the study.

### Participants

To be included, patients had to indicate that they experienced cognitive dysfunction to their general practitioner, or through responding yes when asked about subjective cognitive impairments in our online registry system (OPR) (Granan et al., 2019). Inclusion criteria thus consisted of having a confirmed diagnosis of either localized/neuropathic pain (PNP) or fibromyalgia (FM), and self-reporting cognitive problems. In cases of suspected PNP or FM, the patients were referred within the clinic to a specialist, either a neurologist (PNP) or a specialist in physical medicine and rehabilitation (FM), who then performed a structured assessment of the disorder according to diagnostic guidelines.

Exclusion criteria were diagnosis or suspicion of ongoing mania, psychosis or suicidal ideation with previous suicidal attempts. Participants were also excluded if suicide attempts or plans of suicide were reported during the project period. It was also an exclusion criterion if participants could not speak fluent Norwegian, were pregnant or unable to use the touch screen used for neuropsychological testing. Finally, participants were excluded if they had any disorder or diagnosis that could otherwise explain any potential cognitive impairment, such as an unrelated stroke or diabetes diagnosis. Chronic pain participants often use medications, so the use of stable analgesic drug medication was allowed at all stages for patients already using such medication as part of their treatment regime.

We screened 225 patients, and 79 patients met the criteria for inclusion in the study.

**FIGURE 1:**
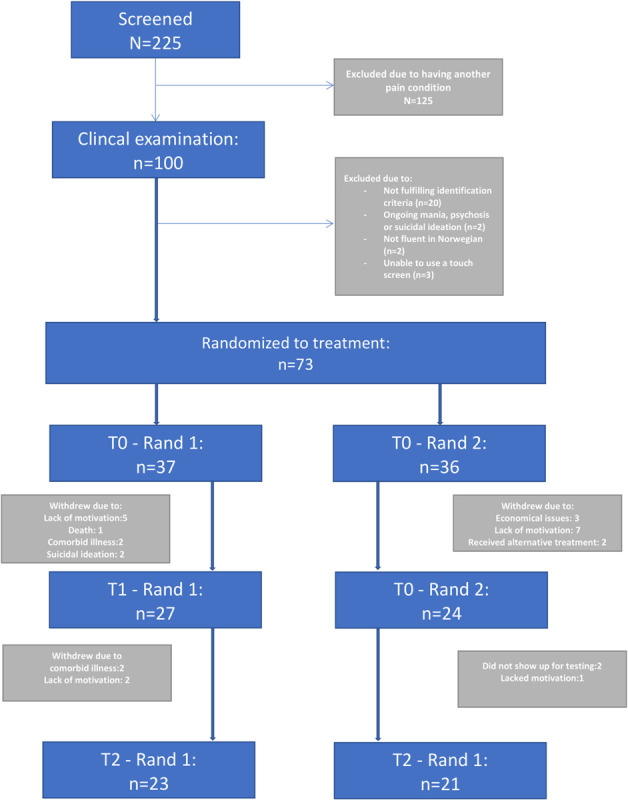
Flow of participants throughout the study.

Of these patients, 73 initiated and completed neuropsychological testing and were available for the current analyses (Figure 1).

### Procedure

At their first visit to the pain clinic, the patients filled out a standardized questionnaire before being examined by either a pain specialist or a complete multidisciplinary pain team (physician, psychologist and occasionally a physiotherapist). All patients met with a clinical psychologist to assess depression and exclusion criteria of mania, psychosis and suicidal ideation. During this examination, patients also filled out an informed consent form and were invited to ask questions about the study. After completing their examination and signing consent, patients received a tablet connected to an online registry system (OPR) (Granan et al., 2019) on which to provide self-report data.

After completing the self-report forms, patients went on to fill out visual analog scales (VAS) and neuropsychological testing within 30 min of completing the online survey. VAS indicated their level of state anxiety and the degree to which they desired to leave the study setting. The VAS was presented as a straight horizontal line of fixed length (100 mm) across a continuum from none to an extreme amount of anxiety or avoidance orientated from the left (none) to the right (extreme). The whole procedure lasted a total of 2.5 h per patient and was repeated three times with a gap of approximately 2 months between each clinical visit. A 1- to 2-weeks’ deviation from the 2-month interval was allowed to facilitate participation.

### Measuring Executive Functions

All participants completed a tailor-made CANTAB cognitive test battery to measure executive functioning. The CANTAB battery is a self-administrated neuropsychological test built for research purposes. It is a widely used cognitive assessment tool (for an overview see e.g., Lenehan et al., 2016) that consists of a series of computerized non-verbal tests. The four tests included in this study provide measures for cognitive functions that have been shown to be impaired in patients with chronic pain in earlier studies, namely the three main components of executive function and visual memory (Miyake and Friedman, 2012). The tests were presented on touchscreen Windows 7 tablet PC running CANTAB-eclipse software (Cambridge, United Kingdom). Participants performed the tests in the following predetermined order, which was then alternated at three times during the study period to protect against order effects: Stop Signal Task (SST), Spatial Working Memory (SWM), Attention Switching Task (AST) and Intra-Extra Dimensional Set Shift (IED), Paired Associates Learning (PAL).

Subjects were seated at a comfortable height in front of the tablet PC that was placed upright on a table using a tablet stand. Instructions were given verbally by the experimenter who followed a standardized test protocol and had full control of a keyboard used to start, pause and terminate each test. Subjects were instructed to carry out the tasks by touching the screen using the forefinger of their dominant hand; this was trained in a “motor screening task” in which they had to touch the center of flashing crosses on the screen.

The current study pre-selected a small set of 10 outcome measures of interest to avoid a blunderbuss approach. These are described below in detail.

#### Primary Outcome - Executive Component of Flexibility

##### Intra-extra dimensional shift

The IED is a computerized analog of the widely used Wisconsin Card Sorting test and is a test of cognitive flexibility (Cambridge Cognition Limited, 2014). In this task, four white-framed boxes were presented on the screen. In each trial, two stimuli (one correct and one incorrect) were presented in two of the boxes. These stimuli were based on two artificial dimensions, color-filled shapes and white lines, and were made up of either one. Feedback teaches the participant which stimulus is correct, and after six correct responses, the stimuli and/or rules are changed. Six consecutive correct responses within 50 trials are required to pass each stage; otherwise, the task ends. The rule for correct responding is modified at each stage to dissociate different aspects of cognitive flexibility. The shifts in correct stimuli are initially intra-dimensional (e.g., within the shape dimension) and then later extra-dimensional, requiring a category shift (e.g., from the shape dimension to the line dimension). The selected outcome IED total errors (IED1), was a composite of the number of completed stages and the number of errors made.

##### Attention switching task (AST)

In this task participants were shown an arrow on the screen that was pointing either to the left or right, and was on either the left or right side of the screen. In the first block (40 assessed trials, 8 practice trials) participants were instructed to respond to the direction of the arrow (and ignore its location). In the second block (40 assessed trials, 8 practice trials) participants were instructed to respond to the location of the arrow (and ignore its direction). In the third and final block (80 assessed trials, 16 practice trials) the instruction randomly switched between responding to the location and the direction of the arrow. In all trials, the location and direction of the arrow could either be congruent (the arrow pointed left and was on the left side of the screen), or incongruent (the arrow pointed left but was on the right side of the screen). Importantly, on incongruent trials, participants had to prevent interference from distracting information (e.g., ignore that the arrow was pointing left when it was on the right side of the screen). On switching trials participants also had to quickly switch the rule they were following without warning (i.e., between responding to the direction and location of the arrow). The main outcome for this test was Switching cost (AST3). Switching cost was the difference between response latencies on switching and non-switching trials.

#### Secondary Outcomes

##### Executive component of inhibitory control

###### Stop signal task (SST)

The SST is a stop signal response inhibition test that measures a subject’s ability to inhibit a prepotent response. In this test, a white ring was presented in the middle of the screen in which an arrow appeared that pointed either to the left or to the right. There were two but-tons on the screen and the subject was told to press the button on the side the arrow points to (for examples of task screens). Some trials (25%) required the subject to withhold the response and not to press the button, signaled by an auditory signal (a beep). The task used a tracking staircase function for the delay between the onset of the visual stimulus and the auditory stop signal (the stop signal delay, SSD): A failed stop trail reduced the subsequent delay by 50 ms and a successful stop increased the delay by 50 ms. This method converged upon an SSD at which the subject successfully stopped approximately 50% of the time (SSD50, calculated using the second half of trials). The tests consisted of five blocks of 64 trials each. At the end of each block, a graph, representing the subject’s performance and a message to the subject (e.g., “Please try to go faster, but do keep stopping when you hear the beep”) was presented to the participants. The outcome variables used in this study was the SSD50, and an estimate of the stop signal reaction time (SSRT) in ms calculated by subtracting the SSD50 from the median reaction time on trials without stopping signal (lower results indicate higher performance). This measure provides a measure of the speed of the inhibitory process (Logan and Cowan, 1984). In addition, we report the proportion of successful stops.

##### Executive component of updating

###### Spatial working memory (SWM)

The SWM test assesses spatial working memory by measuring a subject’s ability to retain spatial information and to manipulate remembered items in working memory (Cambridge Cognition Limited, 2014). In this test, three, four, six or eight colored boxes (depending on the stage) were displayed on the screen. Subjects had to use a process of elimination to find a blue token hidden inside the boxes. They had to touch each box in turn until one opened with a blue token inside. After finding a blue token in one of the boxes, this box would not contain a blue token again. This procedure continued until a blue token had been found in all boxes on the current screen. The test consisted of four practice trials and 12 trials that were recorded. Touching a box in which a blue token had already been found was an error. The outcome measure in this study was a component score reflecting the strategy participants’ used to avoid unnecessary errors.

###### Paired associates learning (PAL)

The PAL test assesses visual memory and new learning (Cambridge Cognition Limited, 2014). Six or eight white boxes (depending on the stage) were displayed on the screen and opened in a randomized order. Depending on the stage, the test presents boxes containing one, two, three, six or eight patterns. After all boxes had been opened the patterns shown in the boxes were displayed in the middle of the screen, one at a time (for examples of task screens). The subject had to touch the box in which the pattern was originally located. At each stage the subjects were given up to ten attempts to get all the locations correct. If the subject could not complete a stage correctly, the test terminated. Total errors adjusted and Total errors 8 shapes adjusted were used as outcome measures. Total errors adjusted reports the number of errors made across all stages with an adjustment for incomplete or failed trials and Total errors 8 shapes adjusted report the numbers of errors made on the last, most difficult stage.

#### Covariates

##### Intelligence testing

The Wechsler Adult Intelligence Scale; (WAIS) IV measures intelligence in adults (Wechsler, 2008). It consists of four index scores attempting to measure four major components of intelligence. As two of the components are strongly correlated with CANTAB tests, namely working memory and processing speed, we chose to use the two components that would add the most to the examination of intelligence in addition to our tests of executive functioning. These were the indexes of verbal comprehension and perceptual reasoning. In the current study, we chose to use the subtests of similarities and matrix reasoning to measure the corresponding indexes.

##### Depression

To determine the presence or absence of *depression* we used the Mini- International Neuropsychiatric Interview 6.0 (M.I.N.I.), a brief structured diagnostic interview for the major Axis I psychiatric disorders in the Diagnostic and Statistical Manual of Mental Disorders (Fifth Edition) and the International Classification of Diseases, Tenth Revision. The M.I.N.I is based on “yes” and “no” answers and covers 16 Axis I disorders and 1 Axis II disorder (antisocial personality disorder; Sheehan et al., 1998).

##### Medication

Participants reported daily medication usage and this was controlled with the list of medications provided by their general practitioner in the referral to the department. Pharmaceuticals were classified by a consensus given by expert physicians as either “Opioids” “Anticonvulsants” or “Antidepressants” based on their active pharmacological ingredients. Morphine equivalents were calculated using a gold standard calculator provided by the centers for disease control^1^ which has been tested for use in a Norwegian pain population.

##### Sleep deficiency

Each participant wore the Philips Respironics Actiwatch Spectrum or Spectrum Pro on the wrist for 7.5 days following the visit to the department. The participants were instructed to press the event marker when getting into bed at night and on waking up in the morning. Data were collected in 15-second epochs. This sampling rate yields 7.5 days. Default sensitivity (medium) was selected since this setting has been found to yield the least overestimation or underestimation of sleep or wakefulness for total sleep time and wake after sleep onset compared to polysomnigraphy. In addition, patients filled out a sleep diary for the same time period yielding a comparable sleep entry should the actigraphy data for some reason not reflect a valid sleep pattern. The Actiwatch and sleep diary were used in combination to create the variables sleep efficiency and average total sleep time.

#### Patient Reported Variables

A complete list of the measures included in the online survey has been published previously as a detailed description of the online registry (OPR) (Granan et al., 2019). To assess pain, we included a series of 0–10 Numeric Rating Scales (NRS) to assess pain intensity (i.e., pain intensity last week) and bothersomeness. The NRS employed an 11-point numerical rating scale with anchors of 0 (“no pain at all”) and 10 (“worst pain possible”). The reliability and validity of the pain NRS is well documented (Granan et al., 2019). The Hopkins Symptom Check List-25 (HSCL-25) was used to assess psychological distress and symptoms of depression. HSCL-25 consists of 25 questions concerning anxiety, depression and somatization, and has been validated in Norwegian. A mean total score of >1.75 is within the normal range, while a score of 1.75 or above indicates psychological distress in need of treatment (Sandanger et al., 1999).

### The MINDFLEX Training Program

The randomized crossover-design was chosen to efficiently estimate effects of active training by within-patient information, in that each patient is his/her own control. Each patient was randomized to either training in the first 8 weeks followed by no training in the next 8 weeks (random = 1), or no training in the first 8 weeks followed by training in the last 8 weeks (random = 2), see Table 1 for data-format. With active training first, a post-active effect can also be assessed (the traditional carryover effect), at the expense of efficiency in estimation of the active effect. With no post-active effect, the crossover design is much more efficient than a parallel-group design with respect to estimation of active training effect (Fitzmaurice et al., 2012).

The participants were given access to a web page on which they could practice on nine different MINDFLEX relational/cognitive flexibility exercises, each employing eight different sets of ideographic stimuli (pain words, non-pain words, positive affect words, and negative affect words, pain images, non-pain images, positive affect images, and negative affect images). The pain word/image stimuli used were based on the patient’s own prior description of pain chosen from a list of Norwegian adjectives describing pain (e.g., burning, tingling, jabbing, pulsating), derived in turn from a dictionary review of pain descriptors by the lead author (HJ).

All training sessions were administered via an internet browser and responses were made either using the left and right arrow keys on a computer keyboard, or via onscreen left and right arrows. Each MINDFLEX administration was approximately 2 min in duration and consisted of two blocks of training with juxtaposed response requirements. That is, one block required participants to produce the same positional (left or right) response for pain word and positive affect stimuli, and non-pain words and negative affect stimuli (i.e., the consistent block). The other block required participants to produce the same positional (left or right) response for pain words and negative affect stimuli, and non-pain words and positive affect stimuli (i.e., the consistent block). Learning during each block was assisted by trial-by-trial feedback and a response window of 3000 ms to enhance fluency. A score was calculated after each pair of randomly sequenced blocks (i.e., one MINDFLEX administration), and presented to the user in the form of a score calculated as (100/[slope of consistent block learning curve – slope of inconsistent block learning curve]). In effect, a high MINDFLEX score represented a small difference in the learning slopes across blocks (high flexibility).

Participants were encouraged to “play” all of the nine MINDFLEX “games” often in their own time over several months with the aim of reaching a target score set by the experimenters for each game. Target scores represented a fluency differential across game blocks that was approaching zero (i.e., near perfect pain stimulus inter-changeability across positional responses classes containing either positive or negative affect stimuli). In effect, the target score attainment requirement was intended to maximize the flexibility of the patients’ responses to pain stimuli as functionally equivalent to positive or negative affect stimuli, depending on current context (i.e., the specific training block requirements). To have some measure of adherence to protocol, we looked at similar CCT in pain patients and concluded with a target of 1,5 h per week of training through 8 weeks as 100% completion. Then a score using number of minutes played gave us groups of 60–79% or 80–100% adherence per protocol when performing subsequent analyses.

### Ethics Statement

The regional committee for medical health and research ethics approved the study (approval number 2016/595) and the protocol was published on www.dam.no (grant number: 2016/FO78689). The study was registered in clinical trials (ClinicalTrials.gov Identifier: NCT02824588).

### Statistical Analyses

Demographics and patient characteristics were analyzed as either number and percent, or mean and standard deviation (SD), our chosen significance level was *p* < 0.05.

An independent statistician who had no part in designing or performing the study performed statistical analysis.

The randomized crossover-design was chosen to efficiently estimate effects of active training by within-patient information. Each patient was randomized to either training in the first 8 weeks followed by no training in the next 8 weeks (random = 1), or no training in the first 8 weeks followed by training in the last 8 weeks (random = 2); see Table 1 for data-format. With active training first, a post-active effect can also be assessed (the traditional carryover effect), at the expense of efficiency in estimation of the active effect. With no post-active effect, the crossover design is much more efficient than a parallel-group design with respect to estimation of active training effect.

#### Model for Learning-Effect

Testing hypothesis 1, complete longitudinal data was taken from each patient’s game-play history with respect to number of training sessions, and number of FAST administrations within each session. A simple linear mixed model with random intercept was used to assess learning effect (1)

$$S\,C\,O\,R\,E_{i\,j\,k}=\beta_{0}+\beta_{1}\,r\,o\,u\,n\,d_{i\,j}+b_{i}+\varepsilon_{i\,j\,k}$$

where *SCORE*_*ijk*_ = |*deviate*_*ijk*_| is the deviation-score (positive or negative) from 0 in a certain game (lower is better), for patient *i* = 1,⋯,*N*, session *j* = 1,⋯,*n*_*i*_ and number of plays *k* = 1,⋯,*n*_*ij*_ within a session, *b*_*i*_ is a random patient-specific intercept and ε_*ijk*_ the error-term.

#### Model for Training-Effect, Crossover-Design

Testing hypothesis 2 and 3, longitudinal analysis of the mean response profiles was performed to impose a minimum of restrictions on the shape of development over time within the two groups, and the covariance between the responses at the three time-points (unstructured covariance). Time was considered as categorical. The model can be described by the following equation:

(2)

$$Y_{i\,j}=\beta_{0}+\beta_{1}\,d\,i\,a\,g_{i}+\beta_{2}\,a\,c\,t\,i\,v\,e_{i\,j}+\beta_{3}\,p\,o\,s\,t\,a\,c\,t\,i\,v\,e_{i\,j}+\gamma_{j}\,t\,i\,m\,e_{j}+\varepsilon_{i\,j}$$

where *Y*_*ij*_ is the outcome for patient *i* = 1,⋯,*N*, at timepoint *j* = 0,1,2. *diag*_*i*_ is a dichotomous indicator for the two diagnostic groups, *active*_*ij*_ and *postactive*_*ij*_ are time-varying indicators of the active training and post-training (only random = 1 group), *time*_*j*_ is categorical indicator for the time-points and *e*_*ij*_ is the error term.

Residuals were inspected to assess model adequacy. With regard to missing data, the estimation method (maximum likelihood) is consistent under the usual missing at random assumption (MAR). All analysis was performed for both the intention-to-treat (ITT) and per-protocol (PP) populations, where the latter was defined as training at least 80% of what was intended (number of minutes registered).

#### Association Between Learning and Cognitive Change

In order to illustrate a potential causal influence between change in game score in one period and change in cognition in the other period, associations were stratified by randomization group. For the rand = 1 group (training in first period) the change in game score in the first period was plotted against cognitive change in the second period. For the rand = 2 group (training in the second period) the cognitive change in the first period was plotted against change in game score in the second period. As a measure of change in game score a linear model, in line with (1) was fitted, without the random effect (each run within person was considered independent trials, except for the individual learning factor). For each person (*i*) the model is given by

(1) $$S\,C\,O\,R\,E_{l}=\alpha +\beta_{i}\,g\,a\,m\,e_{l}+\varepsilon_{l}$$

where *SCORE*_*l*_ = |*deviate*_*l*_| is the deviation-score (positive or negative) from 0 in a certain game (lower is better), for patient *i* = 1,⋯,*N*, game *l* = 1,⋯,*m*_*i*_ (*m*_*i*_ = *n*_*i*_×*n*_*ij*_ from above) and β_*i*_ is the individual “learning slope” and ε_*l*_ the usual error-term.

The individual learning was defined from model (2) as:

(2) $$L\,e\,a\,r\,n\,i\,n\,g_{i}=\hat{S\,C\,O\,R\,E_{1}}-\hat{S\,C\,O\,R\,E_{m_{i}}}$$

Where $\hat{S\,C\,O\,R\,E_{1}}$ and $\hat{S\,C\,O\,R\,E_{m_{i}}}$ are the first and last fitted values from model (2). This individual “Learning” is a quantification of the change in game score, and is plotted against cognitive change (cognition was measured at three time points (*T*_*0*_, *T*_1_,*T*_2_). For the rand = 1 group *Learning*_*i*_ is plotted against the change in cognition from *T*_*1*_ to *T*_*2*_ (*Cogn*_*T*_1__−*Cogn*_*T*_2__). For the rand = 2 group, change in cognition from *T*_*0*_ to *T*_*1*_ (*Cogn*_*T*_0__−*Cogn*_*T*_1__) is plotted against *Learning*_*i*_ (Figure 6).

For the rand = 1 group, with a causal effect of learning in the first period on cognitive change in the second a positive association is expected (higher rate of learning followed by larger positive cognitive change). For the rand = 2 group, a reverse causal effect of cognitive change in the first period on learning in the second period is expected (larger positive cognitive change followed by a higher rate of learning).

All statistical analysis was performed with the statistical package IBM SPSS, version 25.

## Results

The participants in this trial were predominantly females (75%), married (44%), with high school education (49%), who were currently out of work (59%). None of the participants reported anxiety or a wish to avoid the neuropsychological testing as indicated on three separate VAS (anxiety, tension, desire to leave) before administering the test (data not shown). An overview of participant flow is given in Figure 1.

Categorical demographic variables are presented in Table 1a. Interval demographic variables are presented in Table 1b.

**TABLE 1a:** Demographics, patient reported outcomes and medication usage^§^ are presented in 73 patients with either fibromyalgia pain or localized and/or localized and/or neuropathic pain (included from 2016 to 2018).

**Categorical variables**	**Fibromyalgia, localized and neuropathic pain**
	*N* (%)
**Sex**	
Men	18 (25)
Females	55 (75)
**Civil status**	
Single	13 (18)
Married/co-inhabitant	37 (51)
Divorced/widowed	9 (12)
**Education**	
Primary/secondary school	3 (4)
High school diploma	30 (41)
College/university less than 4 years	25 (34)
College/university 4 years or more	3 (4)
**Work status**	
Not working	32 (44)
Working	29 (40)
**Comorbid diagnoses**	
Depression	11 (15)
**Medication usage**	
Opioids (alone)	6 (8)
Anticonvulsants (alone)	7 (10)
Antidepressants (alone)	11 (15)
Opioids + anticonvulsants	4 (5)
Opioids + antidepressants	3 (4)
Anticonvulsants + antidepressants	7 (10)
No medication	29 (40)

*^§^*N* does not always equal 73 on all variables due to missing data, or participants responding with “not applicable.”*

**TABLE 1b:** Demographics and patient reported outcomes are presented in 73 patients with either fibromyalgia pain or localized and/or neuropathic pain (included from 2016 to 2018).

	**Fibromyalgia *N* = 43**	**Localized and/or neuropathic pain *N* = 30**
**Interval**	**Mean (SD)**	**Mean (SD)**
Age	48.5 (10.9)	43.3 (11.4)
Pain intensity^§^	6.8 (1.6)	6.3 (1.8)
Pain bothersomeness^§^	7.0 (1.7)	7.2 (2.1)
Years lived with pain	7.7 (7.0)	17.4 (7.4)
Psychological distress (0–4)^§^	2.1 (0.4)	2.2 (0.5)
Insomnia severity (0–28)^§^	13.7 (6.1)	17.4 (7.4)
Oswestry Disability index (0–50)^§^	34.77 (11.02)	34.21 (11.0)
Fatigue (0–11)^§^	8.06 (3.0)	8.0 (2.8)
Verbal IQ	22.7 (5.1)	24.4 (5.2)
Performance IQ	17.6 (4.8)	18.0 (5.5)
Sleep efficiency %	80.60 (8.93)	79.09 (8.20)
Averaged sleep (hours)	6.88 (1.48)	7.34 (1.27)

*^§^Higher is worse.*

### Establishing a Learning Effect

To evaluate H_1_, whether the MINDFLEX training had a learning effect, the model in (1) was fitted for an arbitrary game (game number 2) with every run within every game play for all patients. A flexibility score was calculated for each run of MINDflex by dividing into 100 the difference in the slope of the consistent block learning curve – the slope of inconsistent block learning curve]). Learning curves slopes represent the rate of increase in fluency (accuracy and speed) over time. This fluency differential score represents increasing fluency as it approaches zero. A slope index of the rate of change in MINDflex scores across time was then calculated. The results of the LMM model with a random intercept, showed a significant negative slope for session, which is interpreted as an improvement (lower deviation from 0) for higher number of sessions (estimate = −0.002, *p* < 0.001, 95% CI: −0.005, −0.001).

### Evaluation of Training Effect on Outcomes Related to Cognitive Inflexibility

To evaluate H_2_ we investigated change on two pre-selected CANTAB outcomes, one of which evaluates switching cost between a given verbal rule and a subsequent stimulus either contradicting this rule or being in line with the given rule. The other outcome evaluates flexibility in terms of rule switching. Participants mean performance and standard deviation on the different time points in the study is presented in Table 2 in the form of raw data from the CANTAB output.

**TABLE 2:** Raw scores on primary outcomes switching cost (AST3) and errors on rule switching (IED1) on the three time points averaged using mean and standard deviation.

**Primary outcome**	N*	**Mean**	**Std. deviation**
Switching cost T1 (AST3)	73	313.50 ms	155.78
Errors on rule switching T1 (IED1)	72	30.25	36.73
Switching cost at T2 (AST3)	52	302.56 ms	163.65
Errors on rule switching T2 (IED1)	51	24.39	18.58
Switching cost T3 (AST3)	44	259.20 ms	160.71
Errors on rule switching T3 (IED1)	43	24.88	29.53

*The reduction in *N* is due to drop-out during follow-up. For both outcomes lower is better.

**FIGURE 2:**
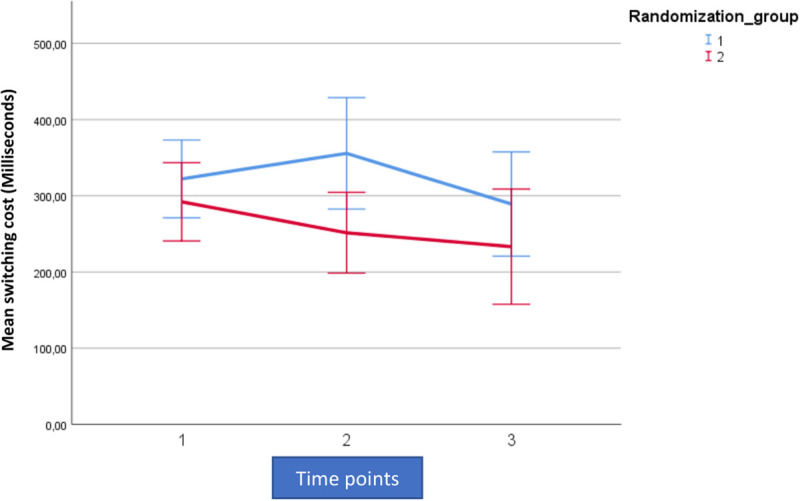
Mean outcome in the two groups (Rand 3 and 2) and the three time points for attention switching cost (AST3) in the trained intention-to-treat population. Time point 1 here equals baseline scores on AST3. Time points 1–2 is training for the first group (Rand:1) while time points 2–3 is training for the second group (Rand:2).

**FIGURE 3:**
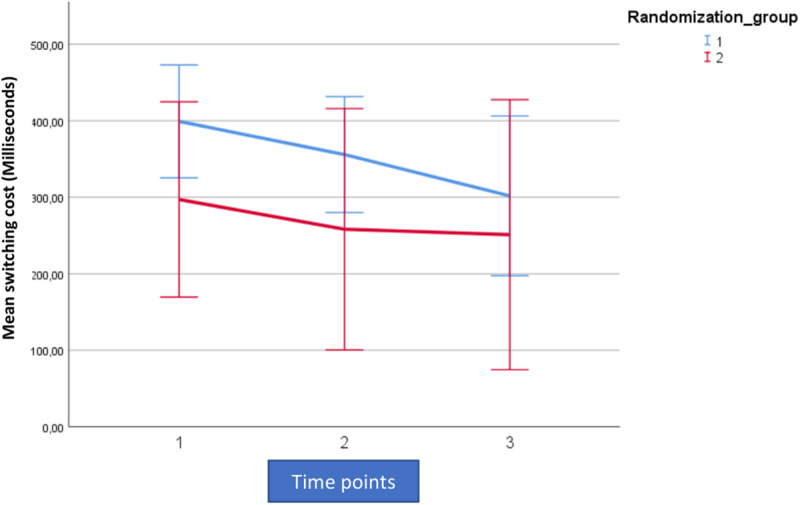
Mean outcome in the two groups (Rand 1 and 2) and the three time points for attention switching cost (AST3) in the trained per-protocol population (80% adherence to training). Time point 1 here equals baseline scores on AST 3. Time points 1–2 is training for the first group (Rand:1) while time points 2–3 is training for the second group (Rand:2).

**FIGURE 4:**
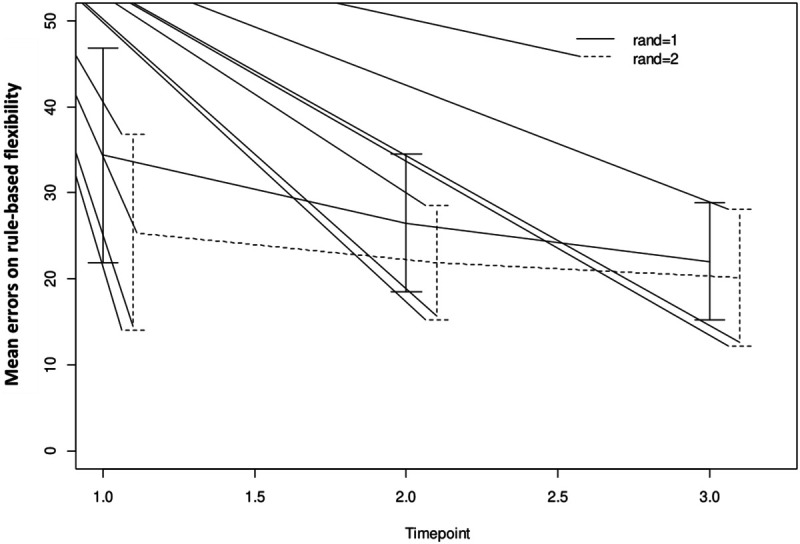
Mean outcome in the two groups and three time points for intra-extra dimensional set shifting (IED1), a rule-based flexibility task. We here show intention-to-treat population (80% adherence to training). Time point 1 here equals baseline scores on IED1. Time points 1–2 is training for the first group (Rand:1) while time points 2–3 is training for the second group (Rand:2). Scaling on the *Y*-axis here indicates total errors on the rule-based flexibility task.

**FIGURE 5:**
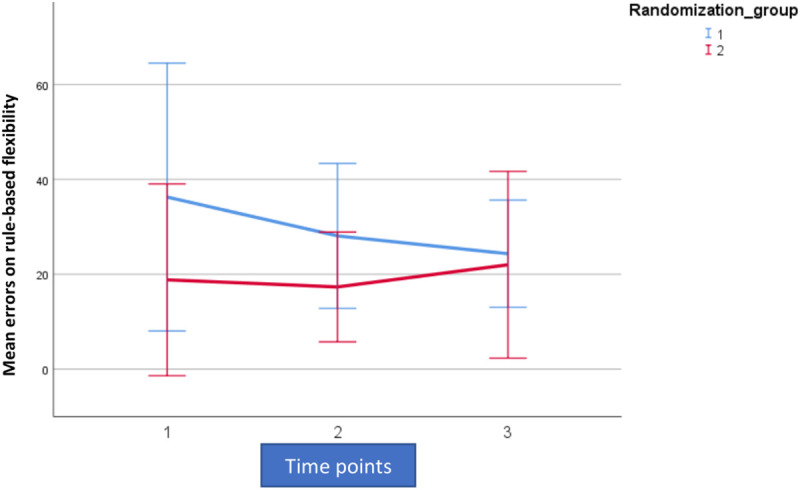
Mean outcome in the two groups and three time points for intra-extra dimensional set shifting (IED1), a rule-based flexibility task. We here show the trained per-protocol population (80% adherence to training). Time point 1 here equals baseline scores on IED1. Time points 1–2 is training for the first group (Rand:1) while time points 2–3 is training for the second group (Rand:2). Scaling on the *Y*-axis here indicates total errors on the rule-based flexibility task.

For analytic purposes the mean outcome for the two groups across the observation period for switching cost (AST3) and errors on rule switching (IED1), for both intent-to-treat- (ITT) and per protocol (PP) populations are shown in Figures 2–5.

For both AST3 and IED1 (lower is better) and both groups, variation is large relative to change over time given the wide confidence intervals shown. The figures suggest a substantial decrease in level (group-average) over the observation period, which is also confirmed in the analysis. However, these time-dependent changes do not reflect being in the active training arm, which is confirmed in our results. Our results showed no significant interaction with diagnostic category (i.e., fibromyalgia or localized pain), but a trend for poorer performance on AST3 was found in the fibromyalgia group (*p* = 0.053).

For AST3 a non-significant effect of active training, in the “wrong” direction was observed in the ITT-population, but this was not the case in the PP-population where an effect in the “correct” direction was observed. The fact that the actual estimated magnitude increased from the ITT- to the PP-population was due to the removal of the non-significant post-treatment effect (Figures 2, 3 and Table 3). The non-significant post-treatment effect led to a statement of insufficient support for H_3_.

**TABLE 3:** Estimated effects of active training and time - effects (results from the linear mixed model).

	**Intent to treat**			**Per protocol (80%)**		
	**Estimate**	**SE**	**95% CI**	**Estimate**	**SE**	**95% CI**
***Attentional switching cost***						
Active training: No	0 (Ref)	.	.	0 (Ref)	.	.
Yes	22,41	20.82	−19,61, 64.43	51.75	27.66	−6.88, 110.39
Time points: 0	0 (Ref)	.		0 (Ref)	.	
1	−20,53	23.35	−67.1, 26.04	−76.38	38.43	−155.72, 2.96
2	−75.8*	21.32	−118.5, −33.09	−97.48*	30.48	−161.16, −33.79
***Rule-based flexibility***						
Active training: No	0 (Ref)	.	.	0 (Ref)	.	.
Yes	1.27	2.66	−4.09, 6.63	5.52	4.78	−4.79, 15.83
Time points: 0	0 (Ref)			0 (Ref)	.	.
1	−5.31	4.43	−14.18, 3.56	−8.82	10.38	−30.58, 12.93
2	−8.7*	4.22	−17.17, −0.22	−9.03	8.96	−27.92, 9.86

The table includes all three time-points (T0–T1–T2), but we have removed non-significant post-training (Rand = 1 reported on post-training effects from T1 to T2) for both the intent-to-treat (ITT) population and those who trained according to protocol (PP). Here T0 is the baseline cognitive testing. *p ≤ 0.05.

Adjusted for the active training effect, the decrease in AST3 level was significant across the observation period, for both ITT- and PP-populations (change = −75.8, 95% CI: −118.5, −33.09) and (change = −97.48, 95% CI: −161.16, −33.79), respectively (Table 3).

For IED1, a significant decrease was found across the observation period in the ITT-population only (change = −8.7, 95% CI: −17.17, −0.22) (Table 3). The reported effect was larger in the PP-population, and the lack of substantial results is a product of low precision.

There was no significant difference between the active and passive training period in the trial and the substantial effects described cannot be attributed to the active training. However, the chosen outcomes of increased flexibility were the only ones that showed any substantial change. None of the other executive function parameters showed any substantial change (Supplementary Appendix 1).

### Additional Analyses

**FIGURE 6:**
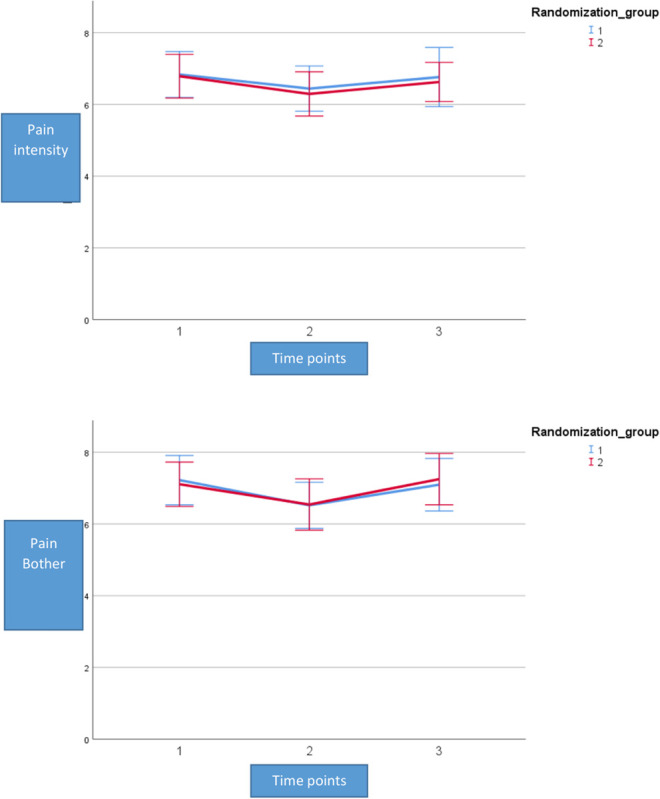
Mean outcome on pain intensity and pain bothersomeness in the two groups (Rand 1 and 2) and the three time points for neuropsychological testing. Time point 1 here equals baseline scores on pain. Time points 1–2 is training for the first group (Rand:1) while time points 2–3 is training for the second group (Rand:2). Pain intensity and pain bothersomeness is reported on a numerical rating scale with the range 0–10.

As this was a pilot study, we also analyzed changes from T0 to T2 on the secondary outcomes of pain and psychological distress. Pain intensity showed the same trajectory as our cognitive flexibility outcomes, where a significant decrease was found across the observation period in the ITT-population, adjusted for an active training effect (change = −0.5, *p* = 0.04, 95% CI: −17.17, −0.22). For pain bothersomeness we also found a significant decrease across the observation period in the ITT-population, adjusted for an active training effect (change = −0.6, *p* = 0.04, 95% CI: −17.17, −0.22). However, none of these effects were directly tied to being in in the active training arm. See Figure 6 for a visualization of the model effects for pain intensity and pain bothersomeness.

No significant effects were found for psychological distress when measured as changes on HSCL-25 and thus data from these analyses is not reported here.

**FIGURE 7:**
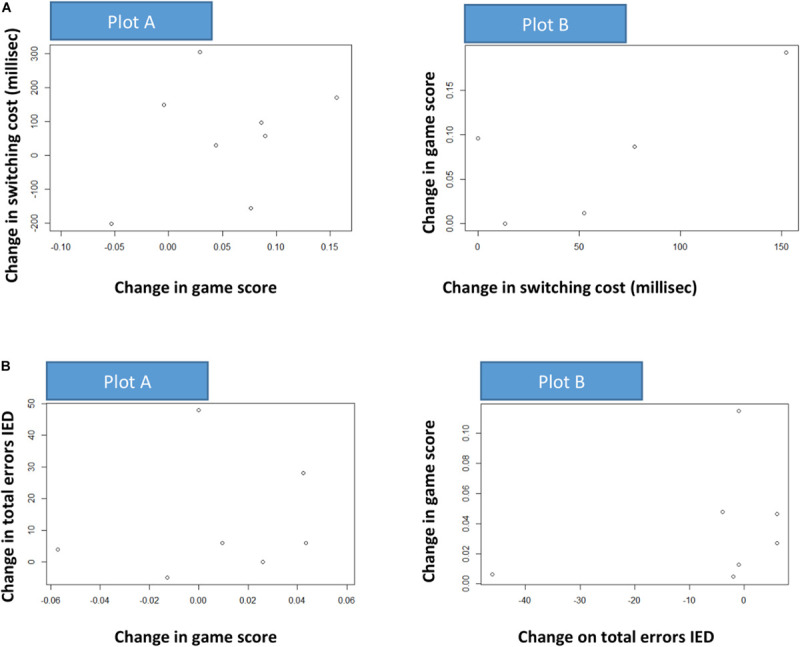
**(A)** This figure illustrates a potential causal influence between change in game score in one period and change in attention switching cost (AST3) in the other period. The dots represent individuals. Plot A (random = 1 group): estimated change (linear model) in fluency on game 2 from T0 to T1 versus change (difference) in attention switching cost from T1 to T2. Plot B (random = 2 group): change in attention switching cost from T0 to T1 versus estimated change (linear model) in fluency on game 2 from T1 to T2. *X*- and *Y*-axis are reversed from A to B. Positive associations are indicated. **(B)** This illustrates a potential causal influence between change in game score in one period and change on rule-based flexibility (IED1) in the other period. The dots represent individuals. Plot A (random = 1 group): estimated change (linear model) in fluency on game 6 from T0 to T1 versus change (difference) in attention switching cost from T1 to T2. Plot B (random = 2 group): change in attention switching cost from T0 to T1 versus estimated change (linear model) in fluency on game 2 from T1 to T2. *X*- and *Y*-axis are reversed from A to B. Positive associations are indicated.

Furthermore, we performed analyses looking at the two games (game 2 and game 6) receiving the most plays over the training period. More specifically, we looked at how changes in learning on games 2 and 6 from T0 to T1 affected changes on our selected primary outcomes. We also looked at how changes in primary outcomes from T1 to T2 related to changes in performance on games 2 and 6. As only a small subset (*n* = 8) of participants improved from time point to time point on both learning and cognitive flexibility, we show absolute numbers of participants illustrated in a scatter plot rather than doing any analyses of significance (Figure 7).

### Summary of Results

When evaluating three hypotheses, the data showed that there was a substantial learning effect from the MINDflex training and a substantial time-dependent improvement on the primary outcomes of increased flexibility, but that this was not due to being in the active training arm. The data showed a non-significant post-treatment effect, which indicated a lack of any significant carry-over effect of the training. Due to large drop-out during the study period, the current data is insufficient to support or reject hypothesis 2 and 3.

## Discussion

We here aimed to pilot the MINDflex training program intended to improve cognitive flexibility in chronic pain patients. The aims of the current study were three-fold, and was evaluated across three hypotheses.

In line with our first hypothesis, we established that the MINDFLEX training program achieved its intended purpose of increasing fluency on both blocks of training. This supports the notion that MINDflex training can engender cognitive defusion as measured by increased fluency across learning tasks that are consistent with or inconsistent with the patient’s verbal history. Due to the large drop-out in the study, the question still remains though if the levels of practice, i.e., time spent training or repetition with the same stimuli, or improving the ideographic selection of words could increase the observed effects.

In support of our second hypothesis we observed a decrease in the chosen CANTAB outcomes from baseline to post-training which was significant, and this decrease in level for both outcomes during the course of the study can be interpreted as improvement in both training-groups. In spite of large variation, small sample-size, and fairly low compliance, a statistically significant improvement was found for the two main outcomes. However, an effect of active training cannot be distinguished from a time/period effect (i.e., that improvement was achieved merely on the basis of inclusion and could not be tied statistically to a causal effect of training).

It is important to note though that only the two pre-selected primary outcomes were improved significantly by the training and none of the other executive functioning measures was substantially affected. This could be viewed as an indication of the training actually transferring to the chosen tasks of cognitive flexibility, but could also be caused by these two outcomes being specifically sensitive to repetitive testing (Rodriguez-Toscano et al., 2020). One explanation that is important is the varying degree of reliability described in CANTAB tests. However, the primary outcome here, the attention switching task, is one of the tests showing a good test-retest reliability in Norwegian adults (Cacciamani et al., 2018; Karlsen et al., 2020).

While we could not tie the substantial effects on our primary outcome causally to the active training, the current data warrants some discussion as well as further research. One avenue of research is looking at improvement of single participants. As we have strictly studied the group as a whole, we do not consider here the fact that some of the participants achieved large effects following training, nor why they appear to do so.

Contrary to our third hypothesis, we did not observe any post-active effects on any of the CANTAB outcomes indicating no carry-over effect from the MINDflex training. This underlines the effect of training being transient. We recognize weaknesses in our data related to only one of the two groups being evaluated with post-active results, resulting in this outcome being difficult to explain *post hoc*. However, in this group there was no indication of a substantial carry-over effect.

Notably, we can report a similar pattern of time effects on the secondary outcomes of pain intensity and pain bothersomeness. This is interesting, because these measurements are inheritably stable and have been shown to be stable over many years in a large Norwegian population study (Landmark et al., 2018). They also tend to show only small improvements in psychological therapies of pain management (Eccleston et al., 2013). The pattern of improvement here could be indicative of our chosen primary outcomes being particularly relevant to pain management.

It is still tempting to speculate regarding the implications of our participants’ improvements, or lack thereof. Trials of cognitive training in different forms show that overall the effects from such training are lacking (Allaire et al., 2014). Particularly when it comes to so-called far transfer of effects.

This lack of effect could be at least in part a task-based problem. It has been argued previously that executive control emerges from dynamic interactions between brain systems mediating language, working memory and attentional processes (Gruber and Goschke, 2004).

Given that the improvements in executive control from attentional and working memory training have been disappointing, we chose to create a training based on an emerging behavioral theory of language and cognition. Brain regions that underlie language functions are also involved in the retrieval and maintenance of verbal goal representations during preparation for task switches. Moreover, context-sensitive behavioral adaptation is linked to the triggering of cognitive control processes that relies on parts of the medial frontal cortex (Gruber and Goschke, 2004).

For pain patients it was therefore possible to envision a far transfer from language based tasks. Explained in brief, expanding the function of words (cognitive de-fusion) could increase flexibility in consequence-driven behavior and rigid rule-governed verbal behavior (Barnes-Holmes and Roche, 2001). As an example, pain patients share implicit attitudes that “pain” is associated with “bad.” Our online digital intervention thus used multi-exemplar operant reinforcement to increase the fluency of patients to respond to “pain”, in some contexts only, as also being “good.” This was to expand the function and increase flexibility of pain-related words, rather than to dismantle the negative associations completely. Such increased flexibility could influence emotional responses and loosen rule-based behavior. Theoretically, cognitive de-fusion should increase the potential for patients to respond to situations appetitively rather than avoidantly using rules-based strategies (Assaz et al., 2018). Even though we could not show a causal role for the increased flexibility associated with the MINDflex game play, we hope to re-create the study with an improved MINDflex training, thereby increasing participant motivation, and reducing dropout rates.

### Limitations

The drop-out rate in this study was the primary limitation. The development of a novel CCT failed to motivate participants sufficiently to participate in the time-consuming longitudinal design of the study. Although subjective and objective tests suggested impairments of executive functioning, the patients were not motivated, or otherwise incapacitated from completing the study to an acceptable degree (see Figure 1).

The MINDflex training should therefore be improved upon with better motivational capacity.

We suggest performing a small qualitative study exploring the reasons for drop-out among participants, and to illuminate any factors that reduced motivation.

## Conclusion

Patients with chronic pain struggle with executive dysfunction and have yet to show improved cognitive capabilities through far transfer of trained skills. The current study examines the potential of harnessing certain RFT concepts to build an intervention for improving executive functioning through a randomized cross-over trial. In accordance with our first hypothesis, we found a learning effect from the program. We also found a significant, positive, change effect over time, but this effect could not be tied to the active training arm.

## Data Availability Statement

The raw data supporting the conclusions of this article will be made available by the authors, without undue reservation.

## Ethics Statement

The studies involving human participants were reviewed and approved by Regional Committees for Health and Medical Ethics. The patients/participants provided their written informed consent to participate in this study.

## Author Contributions

HJ and BR wrote the manuscript. OK created tables and figures. TS and NL critically reviewed the manuscript. All authors contributed to the article and approved the submitted version.

## Conflict of Interest

The authors declare that the research was conducted in the absence of any commercial or financial relationships that could be construed as a potential conflict of interest.

## Footnotes

1 www.cdc.gov
