# Supplementary material for: MINDflex Training for Cognitive Flexibility in Chronic Pain: A Randomized, Controlled Cross-Over Trial
Source: Front Psychol. 2020 Dec 21;11:604832. doi: 10.3389/fpsyg.2020.604832 (PMC7779603; doi:10.3389/fpsyg.2020.604832)
Supplement: Supplementary file 1 [file Table_1.DOCX]

# Plots of the I-T-T population


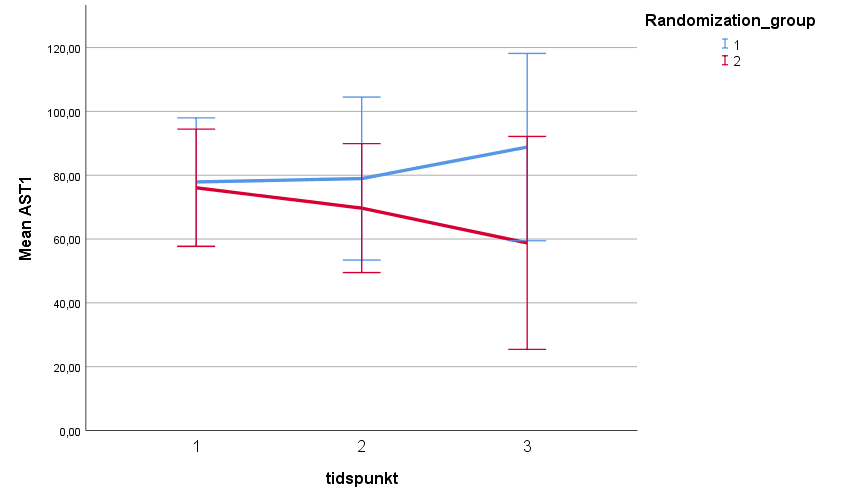


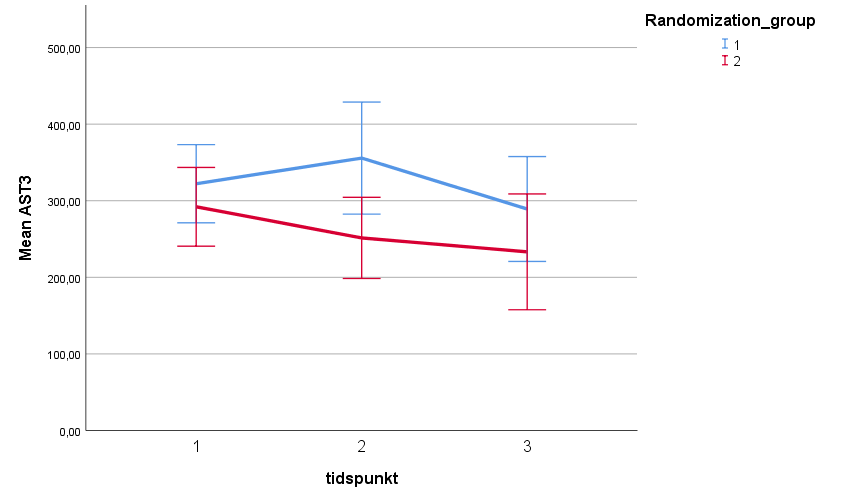


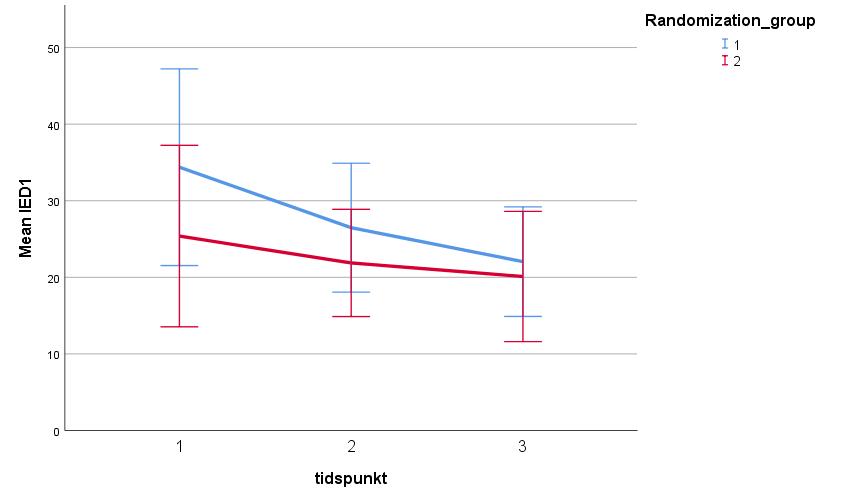


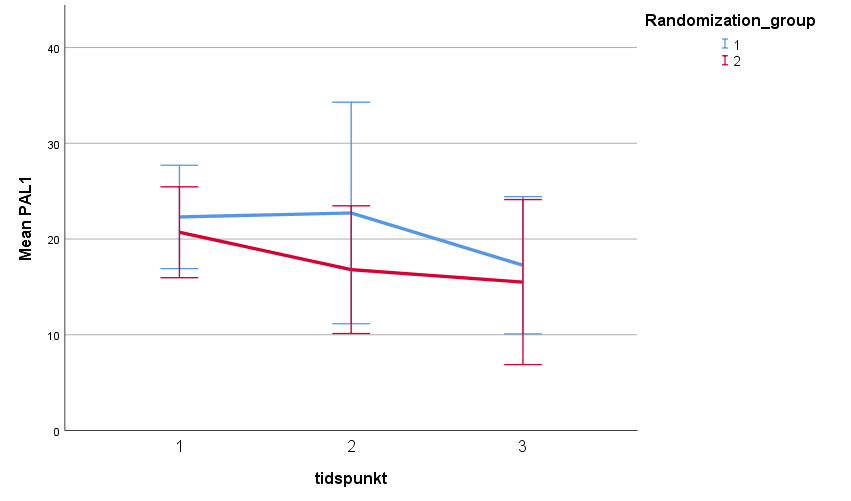


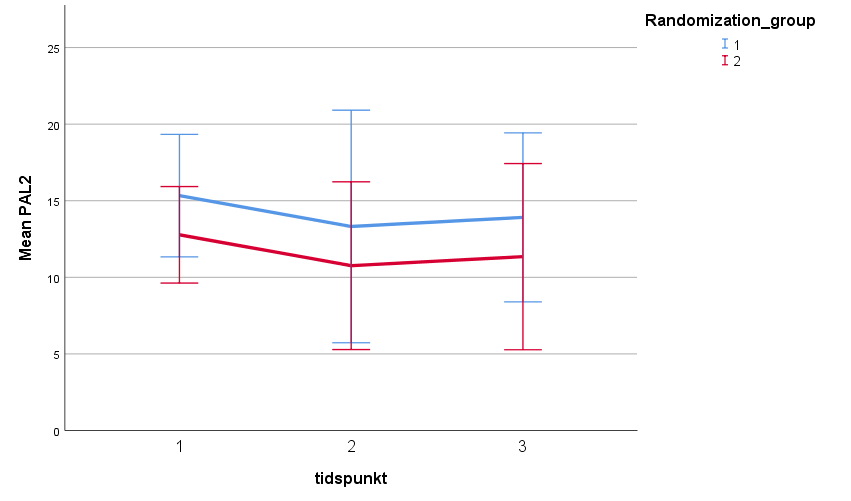


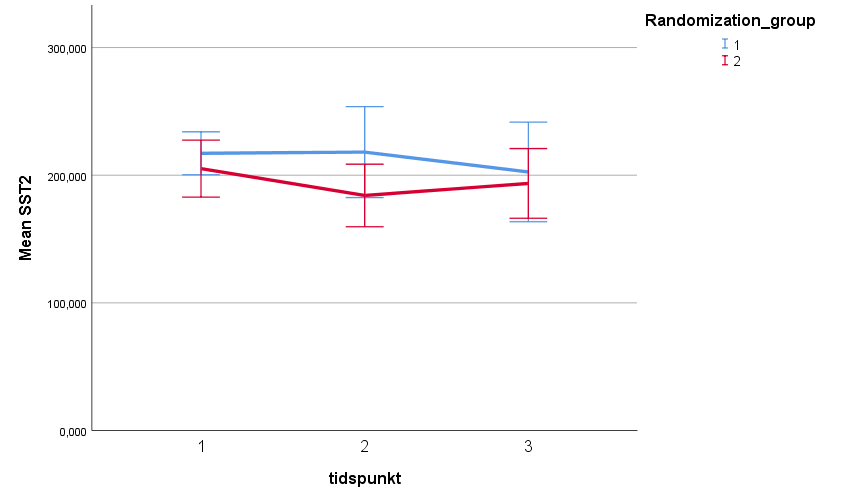


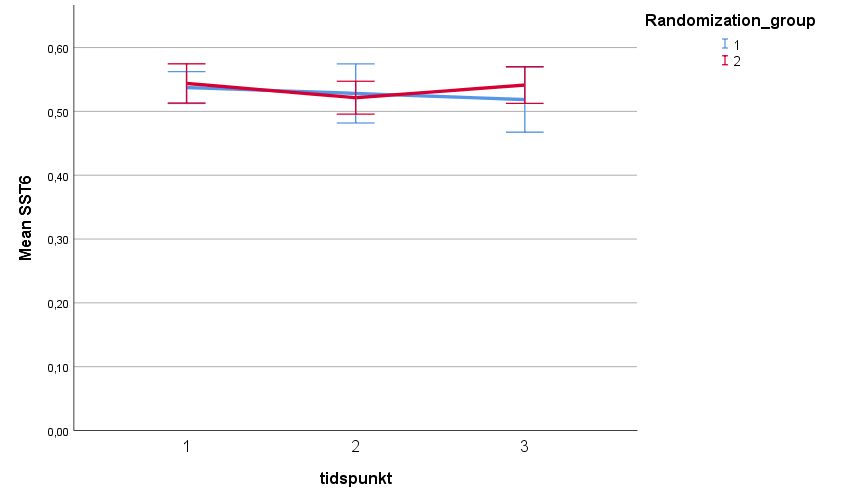


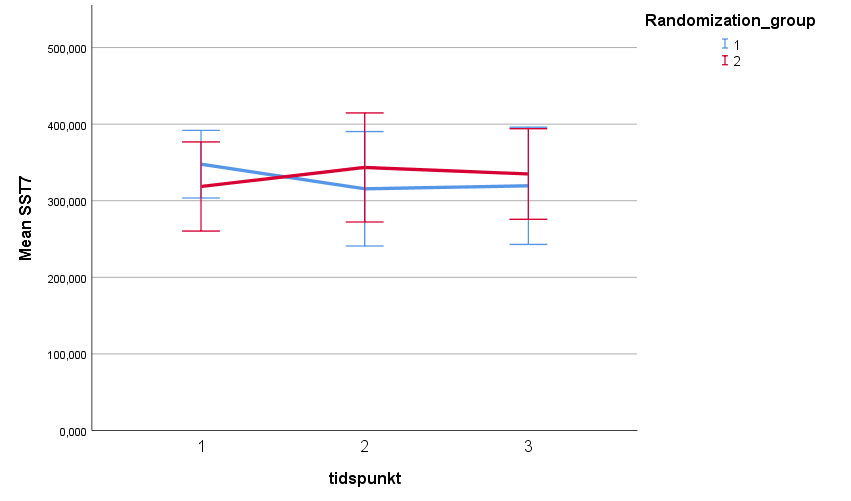


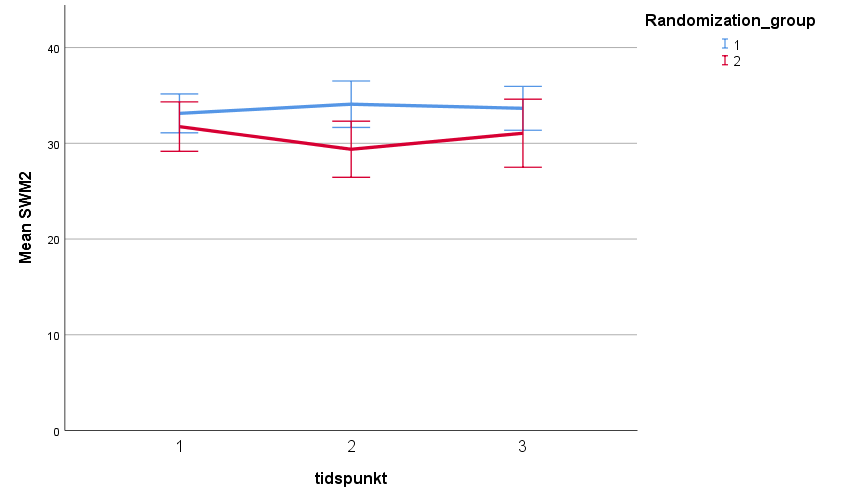


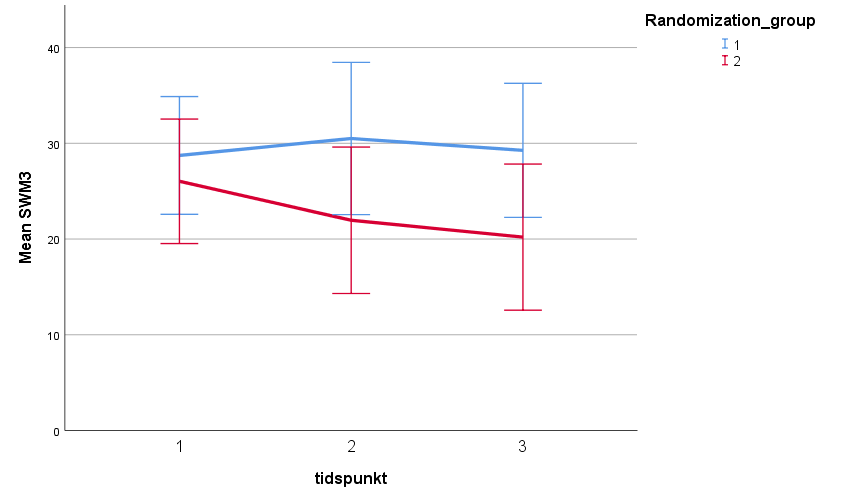


# Plots of the 80 % adherence population


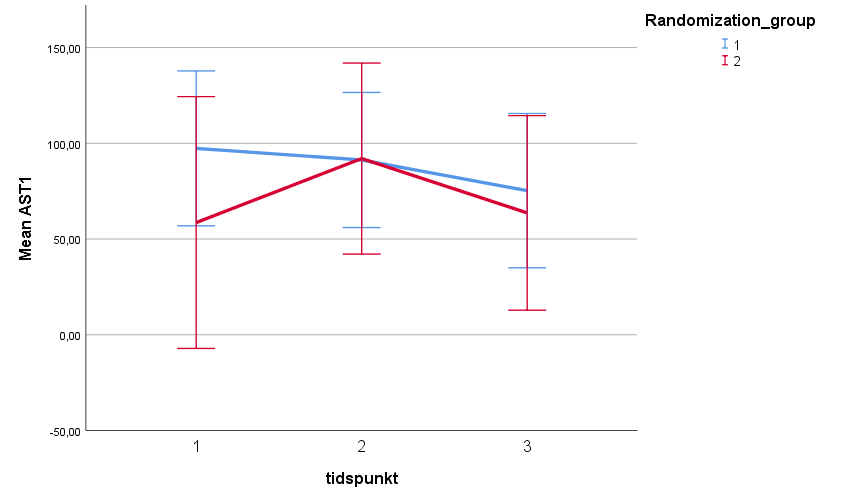


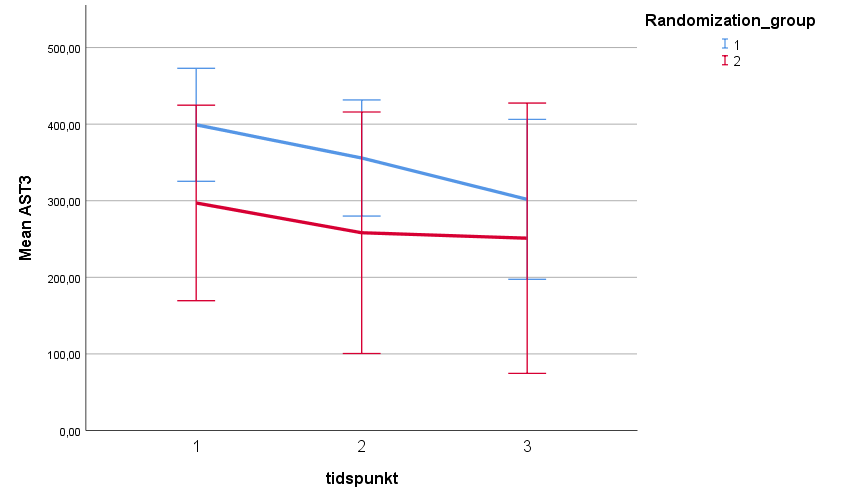


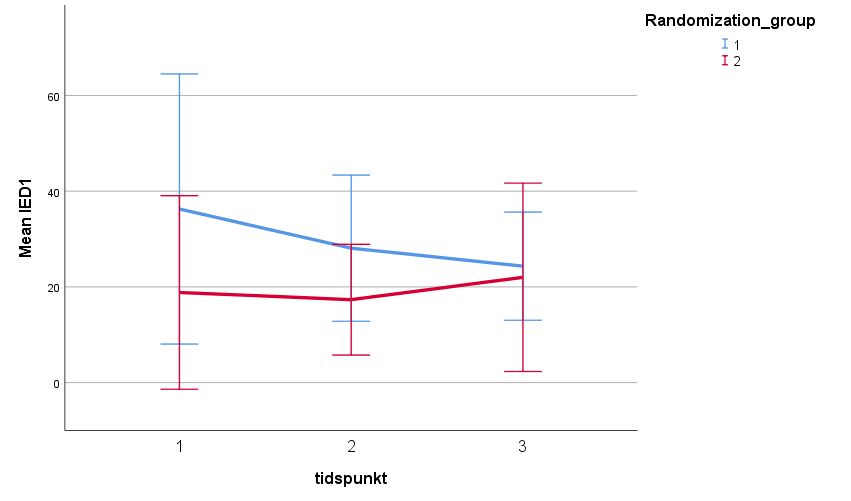


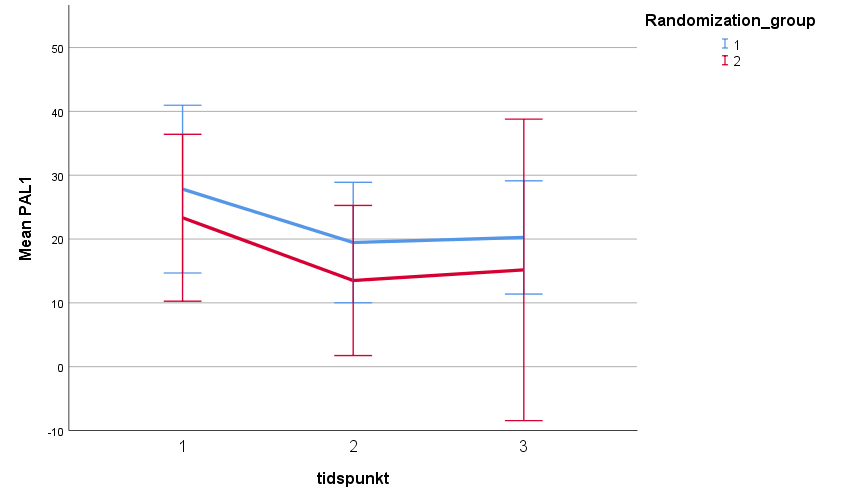


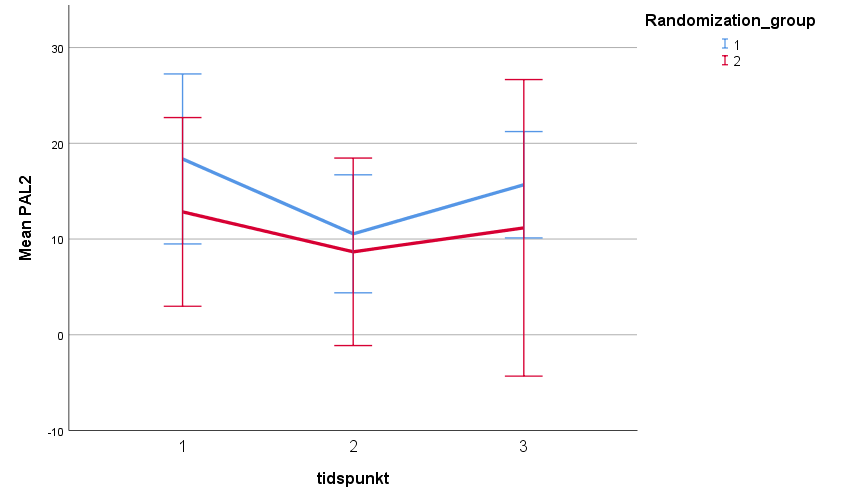


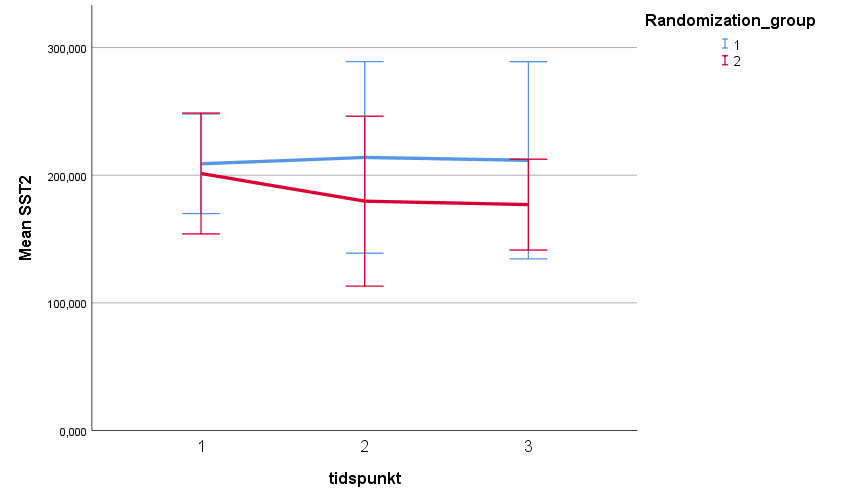


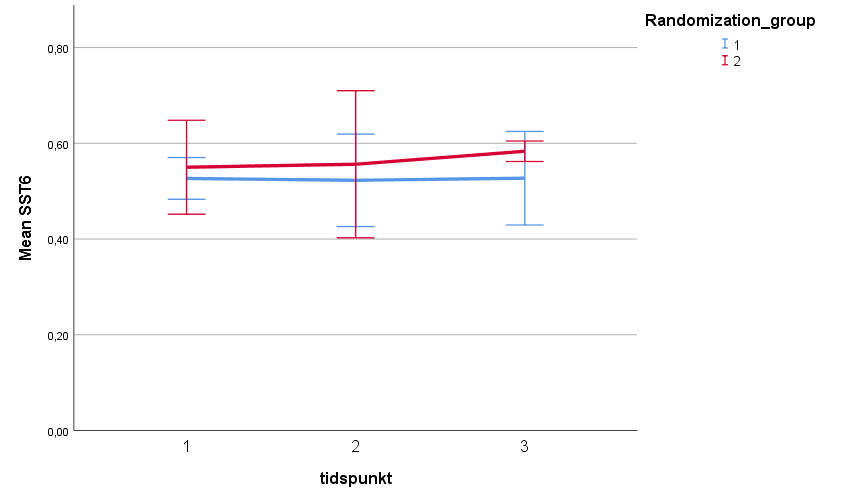


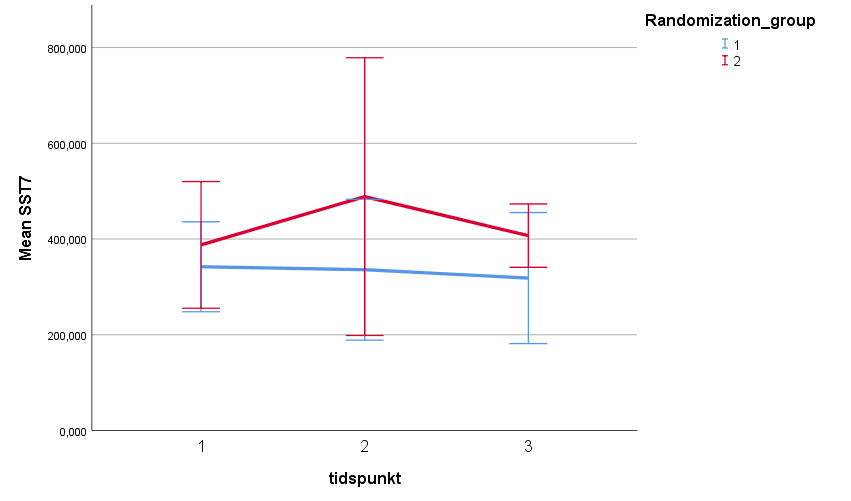


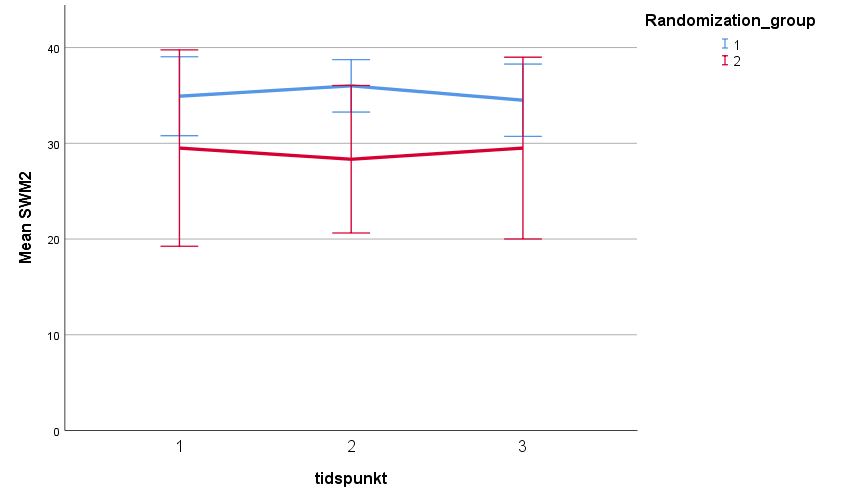


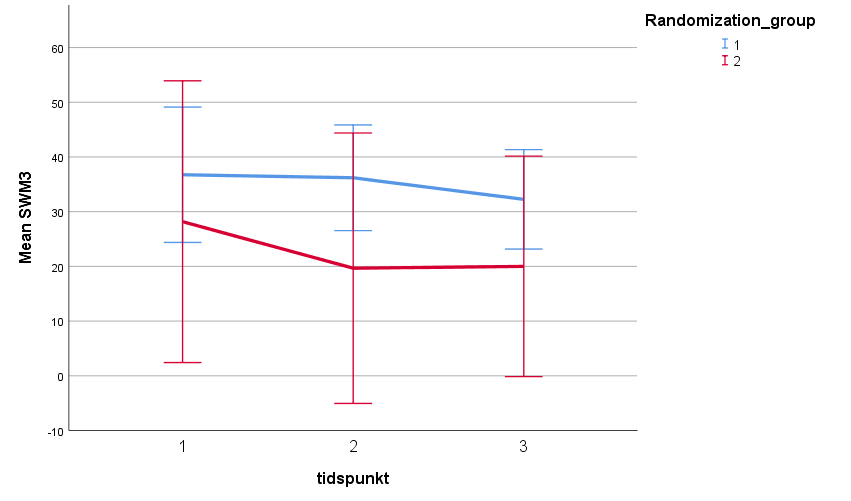


# Plots of the per protocol population 60 % training


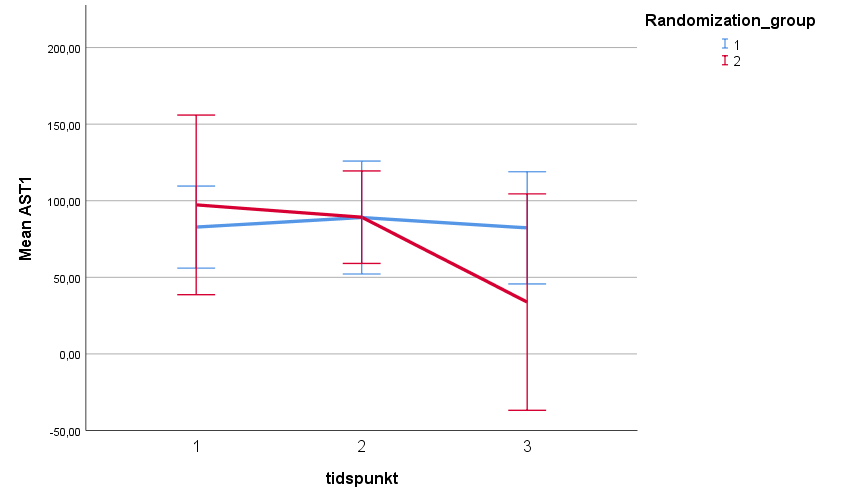


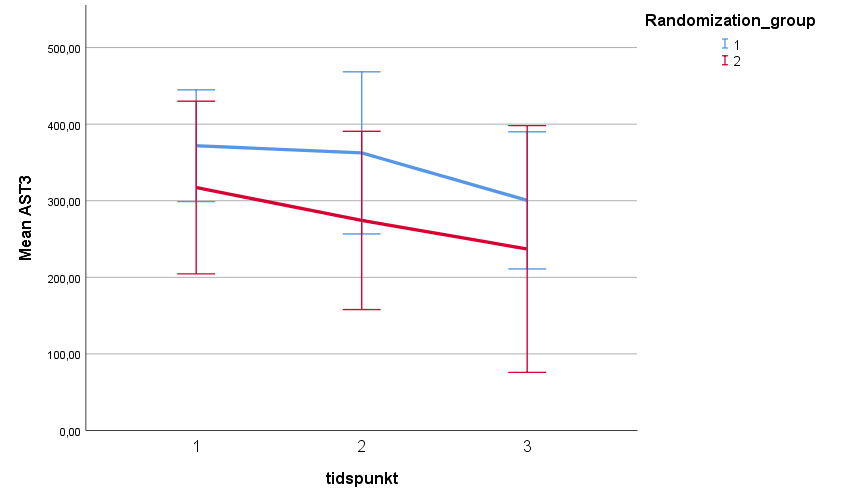


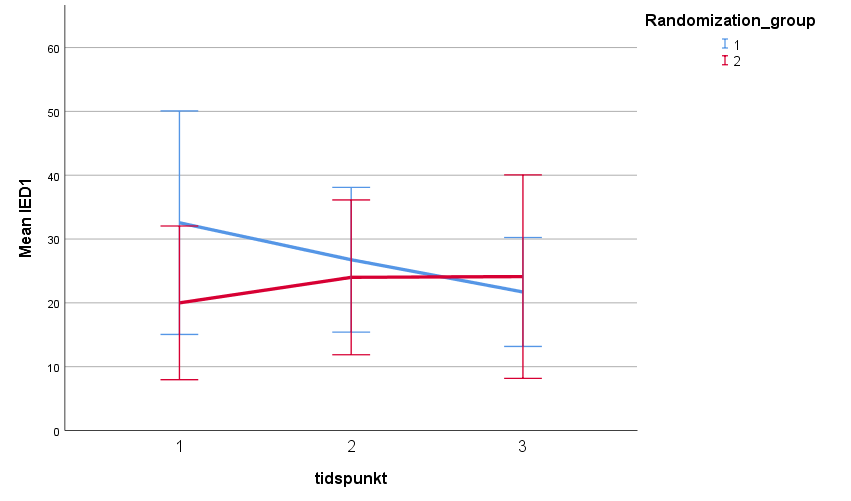


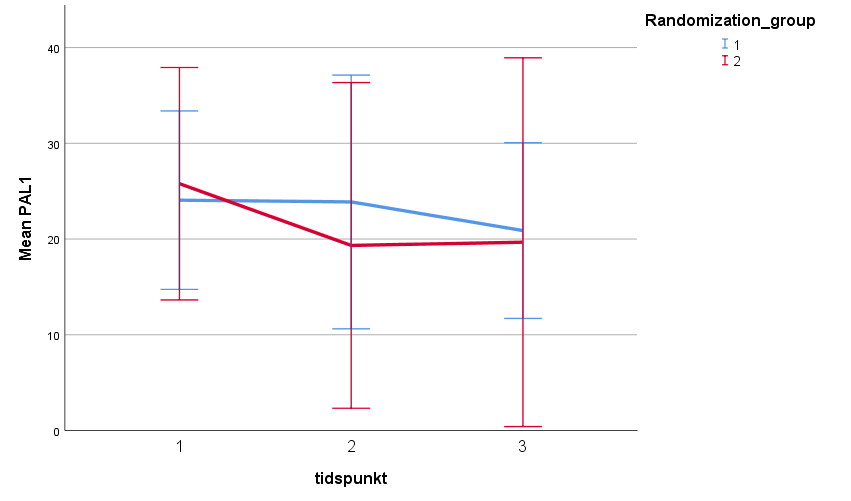


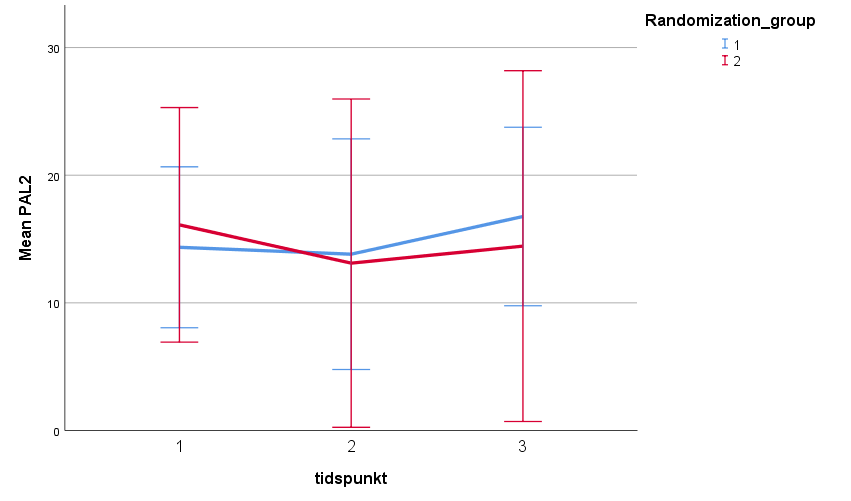


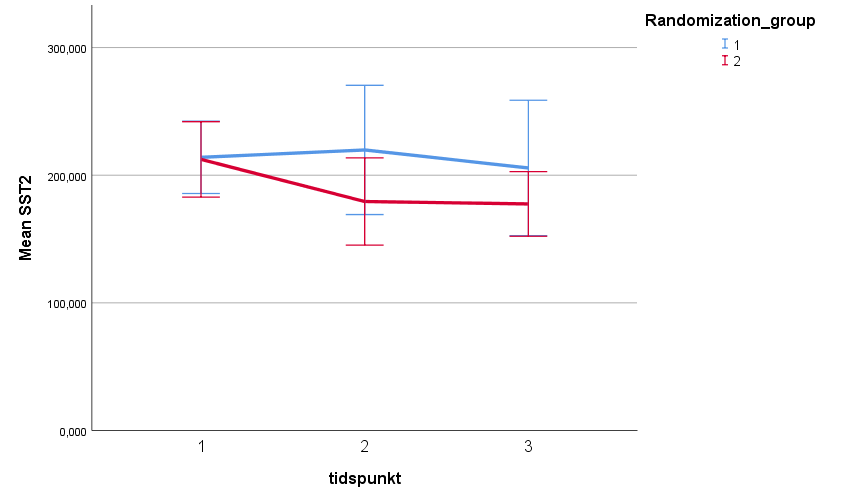


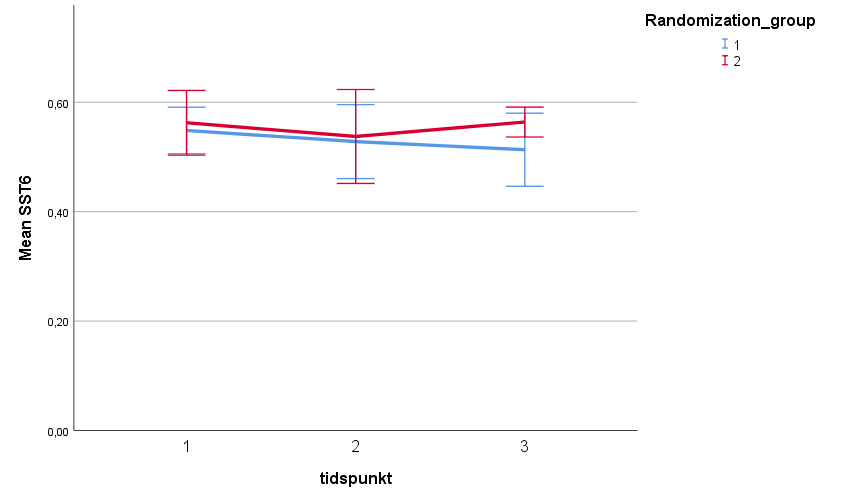


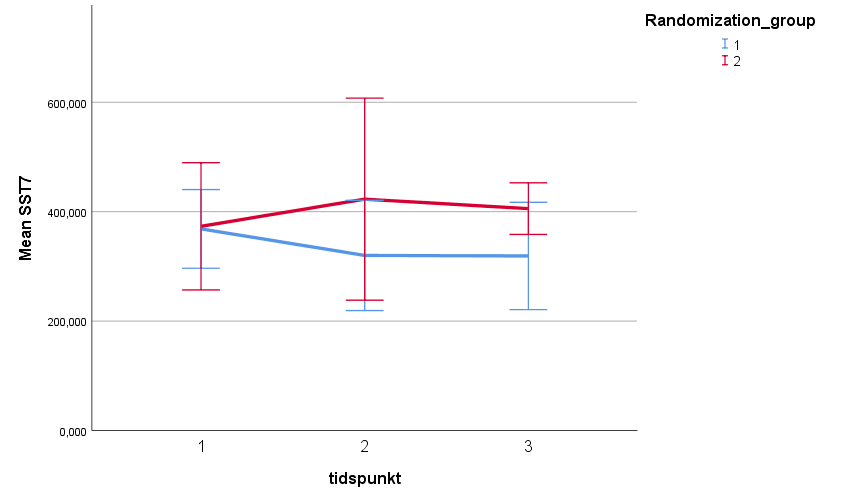


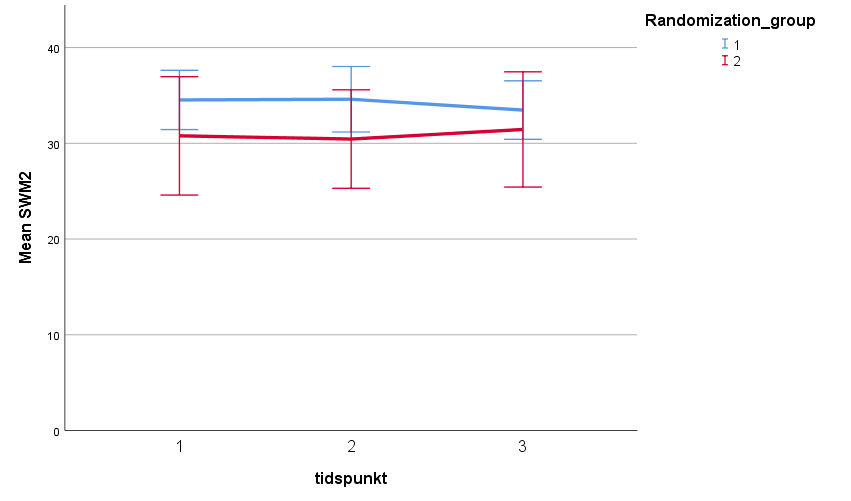


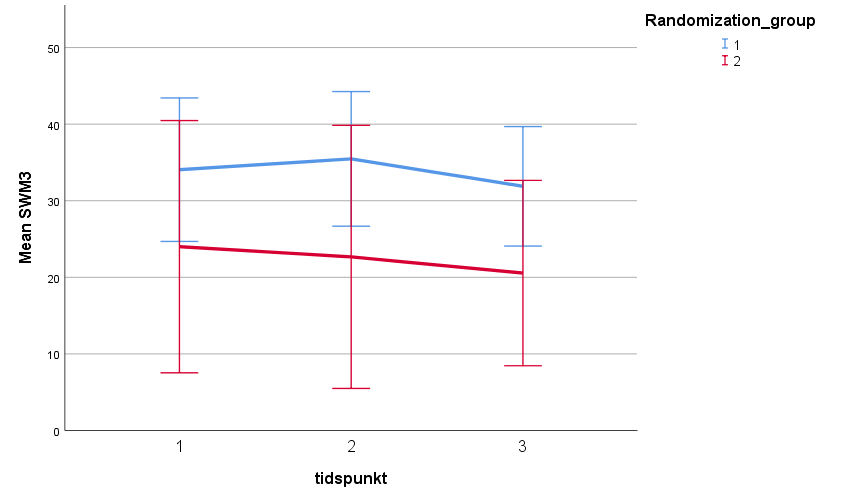


# AST1

## ITT

| **Estimates of Fixed Effects^a^** | | | | | | | |
| --- | --- | --- | --- | --- | --- | --- | --- |
| Parameter | Estimate | Std. Error | df | t | Sig. | 95% Confidence Interval | |
|  |  |  |  |  |  | Lower Bound | Upper Bound |
| Intercept | 77,091237 | 6,813179 | 71,000 | 11,315 | ,000 | 63,506143 | 90,676331 |
| [tidspunktR=1,00] | -35,360471 | 24,920181 | 57,445 | -1,419 | ,161 | -85,253898 | 14,532955 |
| [tidspunktR=2,00] | -8,869317 | 11,495666 | 60,144 | -,772 | ,443 | -31,862943 | 14,124309 |
| [tidspunktR=3,00] | 0^b^ | 0 | . | . | . | . | . |
| trt | 6,636304 | 14,808016 | 49 | ,448 | ,656 | -23,121517 | 36,394126 |
| post_trt | 41,107060 | 30,539580 | 45,208 | 1,346 | ,185 | -20,395005 | 102,609125 |
| a. Dependent Variable: AST1. | | | | | | | |
| b. This parameter is set to zero because it is redundant. | | | | | | | |

Diagnose

| **Estimates of Fixed Effects^a^** | | | | | | | |
| --- | --- | --- | --- | --- | --- | --- | --- |
| Parameter | Estimate | Std. Error | df | t | Sig. | 95% Confidence Interval | |
|  |  |  |  |  |  | Lower Bound | Upper Bound |
| Intercept | 69,001611 | 10,696385 | 78,008 | 6,451 | ,000 | 47,706777 | 90,296446 |
| [tidspunktR=1,00] | -40,105409 | 23,922403 | 57,491 | -1,676 | ,099 | -88,000326 | 7,789509 |
| [tidspunktR=2,00] | -10,050938 | 11,471870 | 59,252 | -,876 | ,384 | -33,004052 | 12,902176 |
| [tidspunktR=3,00] | 0^b^ | 0 | . | . | . | . | . |
| trt | -16,732279 | 20,284545 | 72,478 | -,825 | ,412 | -57,164224 | 23,699665 |
| post_trt | 7,571481 | 36,277512 | 58,218 | ,209 | ,835 | -65,040053 | 80,183015 |
| [Diagnosis=1] | 12,943401 | 13,172400 | 78,987 | ,983 | ,329 | -13,275674 | 39,162476 |
| [Diagnosis=2] | 0^b^ | 0 | . | . | . | . | . |
| [Diagnosis=1] * trt | 34,476631 | 19,829990 | 96,562 | 1,739 | ,085 | -4,882666 | 73,835928 |
| [Diagnosis=2] * trt | 0^b^ | 0 | . | . | . | . | . |
| [Diagnosis=1] * post_trt | 52,324855 | 30,648630 | 47,778 | 1,707 | ,094 | -9,305754 | 113,955465 |
| [Diagnosis=2] * post_trt | 0^b^ | 0 | . | . | . | . | . |
| a. Dependent Variable: AST1. | | | | | | | |
| b. This parameter is set to zero because it is redundant. | | | | | | | |

## PP, (80 % adherence)

| **Estimates of Fixed Effects^a^** | | | | | | | |
| --- | --- | --- | --- | --- | --- | --- | --- |
| Parameter | Estimate | Std. Error | df | t | Sig. | 95% Confidence Interval | |
|  |  |  |  |  |  | Lower Bound | Upper Bound |
| Intercept | 84,421843 | 15,145045 | 17,000 | 5,574 | ,000 | 52,468591 | 116,375095 |
| [tidspunktR=1,00] | 2,348268 | 42,632783 | 18,628 | ,055 | ,957 | -87,003911 | 91,700447 |
| [tidspunktR=2,00] | 17,768782 | 21,250001 | 20,039 | ,836 | ,413 | -26,552372 | 62,089936 |
| [tidspunktR=3,00] | 0^b^ | 0 | . | . | . | . | . |
| trt | -15,997955 | 23,470826 | 16,000 | -,682 | ,505 | -65,753884 | 33,757974 |
| post_trt | -15,068448 | 47,650730 | 16,000 | -,316 | ,756 | -116,083484 | 85,946588 |
| a. Dependent Variable: AST1. | | | | | | | |
| b. This parameter is set to zero because it is redundant. | | | | | | | |

| **Estimates of Fixed Effects^a^** | | | | | | | |
| --- | --- | --- | --- | --- | --- | --- | --- |
| Parameter | Estimate | Std. Error | df | t | Sig. | 95% Confidence Interval | |
|  |  |  |  |  |  | Lower Bound | Upper Bound |
| Intercept | 69,901951 | 23,576027 | 18,833 | 2,965 | ,008 | 20,527160 | 119,276742 |
| [tidspunktR=1,00] | ,201862 | 38,942759 | 15,239 | ,005 | ,996 | -82,689595 | 83,093319 |
| [tidspunktR=2,00] | 16,920064 | 19,806085 | 15,625 | ,854 | ,406 | -25,149065 | 58,989193 |
| [tidspunktR=3,00] | 0^b^ | 0 | . | . | . | . | . |
| trt | -22,089855 | 26,085365 | 18,548 | -,847 | ,408 | -76,777321 | 32,597612 |
| post_trt | -24,472588 | 47,816278 | 16,635 | -,512 | ,616 | -125,524922 | 76,579746 |
| [Diagnosis=1] | 23,759824 | 28,131598 | 18,574 | ,845 | ,409 | -35,211763 | 82,731411 |
| [Diagnosis=2] | 0^b^ | 0 | . | . | . | . | . |
| [Diagnosis=1] * trt | 12,625674 | 24,991723 | 27,990 | ,505 | ,617 | -38,568337 | 63,819686 |
| [Diagnosis=2] * trt | 0^b^ | 0 | . | . | . | . | . |
| [Diagnosis=1] * post_trt | 19,647670 | 34,737231 | 16,093 | ,566 | ,579 | -53,957485 | 93,252824 |
| [Diagnosis=2] * post_trt | 0^b^ | 0 | . | . | . | . | . |
| a. Dependent Variable: AST1. | | | | | | | |
| b. This parameter is set to zero because it is redundant. | | | | | | | |

## PP (60 % adherence)

| **Estimates of Fixed Effects^a^** | | | | | | | |
| --- | --- | --- | --- | --- | --- | --- | --- |
| Parameter | Estimate | Std. Error | df | t | Sig. | 95% Confidence Interval | |
|  |  |  |  |  |  | Lower Bound | Upper Bound |
| Intercept | 87,449737 | 11,699795 | 27,000 | 7,474 | ,000 | 63,443741 | 111,455732 |
| [tidspunktR=1,00] | -56,700945 | 45,467079 | 28,382 | -1,247 | ,223 | -149,779604 | 36,377713 |
| [tidspunktR=2,00] | -1,317744 | 21,342985 | 28,922 | -,062 | ,951 | -44,974167 | 42,338680 |
| [tidspunktR=3,00] | 0^b^ | 0 | . | . | . | . | . |
| trt | 3,184148 | 24,483177 | 24,000 | ,130 | ,898 | -47,346646 | 53,714941 |
| post_trt | 51,540253 | 51,292342 | 24,000 | 1,005 | ,325 | -54,321938 | 157,402444 |
| a. Dependent Variable: AST1. | | | | | | | |
| b. This parameter is set to zero because it is redundant. | | | | | | | |

| **Estimates of Fixed Effects^a^** | | | | | | | |
| --- | --- | --- | --- | --- | --- | --- | --- |
| Parameter | Estimate | Std. Error | df | t | Sig. | 95% Confidence Interval | |
|  |  |  |  |  |  | Lower Bound | Upper Bound |
| Intercept | 99,957666 | 20,975040 | 29,060 | 4,766 | ,000 | 57,062761 | 142,852571 |
| [tidspunktR=1,00] | -57,387996 | 43,412264 | 27,403 | -1,322 | ,197 | -146,401407 | 31,625415 |
| [tidspunktR=2,00] | -2,909188 | 20,457282 | 27,170 | -,142 | ,888 | -44,871777 | 39,053401 |
| [tidspunktR=3,00] | 0^b^ | 0 | . | . | . | . | . |
| trt | -40,470086 | 31,042986 | 33,537 | -1,304 | ,201 | -103,589138 | 22,648966 |
| post_trt | 2,890101 | 57,843029 | 29,819 | ,050 | ,960 | -115,271132 | 121,051335 |
| [Diagnosis=1] | -17,511101 | 24,370138 | 29,401 | -,719 | ,478 | -67,324098 | 32,301896 |
| [Diagnosis=2] | 0^b^ | 0 | . | . | . | . | . |
| [Diagnosis=1] * trt | 64,118306 | 28,392459 | 44,910 | 2,258 | ,029 | 6,929796 | 121,306816 |
| [Diagnosis=2] * trt | 0^b^ | 0 | . | . | . | . | . |
| [Diagnosis=1] * post_trt | 69,803497 | 43,647176 | 28,903 | 1,599 | ,121 | -19,477982 | 159,084976 |
| [Diagnosis=2] * post_trt | 0^b^ | 0 | . | . | . | . | . |
| a. Dependent Variable: AST1. | | | | | | | |
| b. This parameter is set to zero because it is redundant. | | | | | | | |

# AST3

## ITT

| **Estimates of Fixed Effects^a^** | | | | | | | |
| --- | --- | --- | --- | --- | --- | --- | --- |
| Parameter | Estimate | Std. Error | df | t | Sig. | 95% Confidence Interval | |
|  |  |  |  |  |  | Lower Bound | Upper Bound |
| Intercept | 309,233133 | 17,974119 | 71 | 17,204 | ,000 | 273,393758 | 345,072509 |
| [tidspunktR=1,00] | -161,218657 | 51,631531 | 55,300 | -3,122 | ,003 | -264,677932 | -57,759382 |
| [tidspunktR=2,00] | -49,753667 | 28,266006 | 55,474 | -1,760 | ,084 | -106,389137 | 6,881803 |
| [tidspunktR=3,00] | 0^b^ | 0 | . | . | . | . | . |
| trt | 80,175614 | 37,698570 | 49 | 2,127 | ,039 | 4,417501 | 155,933728 |
| post_trt | 111,514569 | 61,054449 | 45,967 | 1,826 | ,074 | -11,384077 | 234,413214 |
| a. Dependent Variable: AST3. | | | | | | | |
| b. This parameter is set to zero because it is redundant. | | | | | | | |

Diagnose

| **Estimates of Fixed Effects^a^** | | | | | | | |
| --- | --- | --- | --- | --- | --- | --- | --- |
| Parameter | Estimate | Std. Error | df | t | Sig. | 95% Confidence Interval | |
|  |  |  |  |  |  | Lower Bound | Upper Bound |
| Intercept | 270,912455 | 28,112339 | 74,920 | 9,637 | ,000 | 214,908830 | 326,916080 |
| [tidspunktR=1,00] | -170,878044 | 52,021288 | 55,079 | -3,285 | ,002 | -275,127655 | -66,628432 |
| [tidspunktR=2,00] | -53,890804 | 28,449244 | 55,122 | -1,894 | ,063 | -110,901519 | 3,119910 |
| [tidspunktR=3,00] | 0^b^ | 0 | . | . | . | . | . |
| trt | 59,116684 | 48,659573 | 73,243 | 1,215 | ,228 | -37,856275 | 156,089643 |
| post_trt | 117,970974 | 73,028014 | 59,768 | 1,615 | ,111 | -28,118455 | 264,060404 |
| [Diagnosis=1] | 61,313085 | 34,950666 | 74,930 | 1,754 | ,083 | -8,313271 | 130,939442 |
| [Diagnosis=2] | 0^b^ | 0 | . | . | . | . | . |
| [Diagnosis=1] * trt | 33,798937 | 43,690681 | 94,172 | ,774 | ,441 | -52,947871 | 120,545745 |
| [Diagnosis=2] * trt | 0^b^ | 0 | . | . | . | . | . |
| [Diagnosis=1] * post_trt | 3,906918 | 55,309503 | 42,402 | ,071 | ,944 | -107,680864 | 115,494700 |
| [Diagnosis=2] * post_trt | 0^b^ | 0 | . | . | . | . | . |
| a. Dependent Variable: AST3. | | | | | | | |
| b. This parameter is set to zero because it is redundant. | | | | | | | |

## PP (80 % adherence)

| **Estimates of Fixed Effects^a^** | | | | | | | |
| --- | --- | --- | --- | --- | --- | --- | --- |
| Parameter | Estimate | Std. Error | df | t | Sig. | 95% Confidence Interval | |
|  |  |  |  |  |  | Lower Bound | Upper Bound |
| Intercept | 365,119174 | 29,360979 | 17 | 12,436 | ,000 | 303,172923 | 427,065425 |
| [tidspunktR=1,00] | -112,952542 | 97,558593 | 16,802 | -1,158 | ,263 | -318,967988 | 93,062905 |
| [tidspunktR=2,00] | -82,492717 | 53,505041 | 18,538 | -1,542 | ,140 | -194,669265 | 29,683831 |
| [tidspunktR=3,00] | 0^b^ | 0 | . | . | . | . | . |
| trt | 60,920352 | 61,344158 | 16 | ,993 | ,335 | -69,123453 | 190,964157 |
| post_trt | 18,632361 | 111,287752 | 16 | ,167 | ,869 | -217,287134 | 254,551857 |
| a. Dependent Variable: AST3. | | | | | | | |
| b. This parameter is set to zero because it is redundant. | | | | | | | |

| **Estimates of Fixed Effects^a^** | | | | | | | |
| --- | --- | --- | --- | --- | --- | --- | --- |
| Parameter | Estimate | Std. Error | df | t | Sig. | 95% Confidence Interval | |
|  |  |  |  |  |  | Lower Bound | Upper Bound |
| Intercept | 344,830897 | 43,529452 | 19,367 | 7,922 | ,000 | 253,839344 | 435,822451 |
| [tidspunktR=1,00] | -109,865896 | 100,441484 | 16,049 | -1,094 | ,290 | -322,739020 | 103,007228 |
| [tidspunktR=2,00] | -82,914431 | 53,935830 | 18,095 | -1,537 | ,142 | -196,186592 | 30,357731 |
| [tidspunktR=3,00] | 0^b^ | 0 | . | . | . | . | . |
| trt | 89,047874 | 70,481102 | 19,915 | 1,263 | ,221 | -58,013332 | 236,109080 |
| post_trt | -13,979683 | 120,968188 | 16,639 | -,116 | ,909 | -269,622360 | 241,662993 |
| [Diagnosis=1] | 33,198999 | 51,747453 | 17,355 | ,642 | ,530 | -75,808665 | 142,206662 |
| [Diagnosis=2] | 0^b^ | 0 | . | . | . | . | . |
| [Diagnosis=1] * trt | -47,134203 | 56,759026 | 29,604 | -,830 | ,413 | -163,116670 | 68,848264 |
| [Diagnosis=2] * trt | 0^b^ | 0 | . | . | . | . | . |
| [Diagnosis=1] * post_trt | 50,793799 | 69,842993 | 15,106 | ,727 | ,478 | -97,982508 | 199,570105 |
| [Diagnosis=2] * post_trt | 0^b^ | 0 | . | . | . | . | . |
| a. Dependent Variable: AST3. | | | | | | | |
| b. This parameter is set to zero because it is redundant. | | | | | | | |

## PP (60 % adherence)

| **Estimates of Fixed Effects^a^** | | | | | | | |
| --- | --- | --- | --- | --- | --- | --- | --- |
| Parameter | Estimate | Std. Error | df | t | Sig. | 95% Confidence Interval | |
|  |  |  |  |  |  | Lower Bound | Upper Bound |
| Intercept | 354,270152 | 28,239330 | 27,000 | 12,545 | ,000 | 296,327833 | 412,212471 |
| [tidspunktR=1,00] | -135,833915 | 87,956346 | 25,972 | -1,544 | ,135 | -316,640421 | 44,972591 |
| [tidspunktR=2,00] | -54,523279 | 53,726561 | 24,889 | -1,015 | ,320 | -165,200290 | 56,153732 |
| [tidspunktR=3,00] | 0^b^ | 0 | . | . | . | . | . |
| trt | 52,713165 | 65,542677 | 24 | ,804 | ,429 | -82,560273 | 187,986602 |
| post_trt | 68,575467 | 97,726017 | 24 | ,702 | ,490 | -133,121120 | 270,272054 |
| a. Dependent Variable: AST3. | | | | | | | |
| b. This parameter is set to zero because it is redundant. | | | | | | | |

| **Estimates of Fixed Effects^a^** | | | | | | | |
| --- | --- | --- | --- | --- | --- | --- | --- |
| Parameter | Estimate | Std. Error | df | t | Sig. | 95% Confidence Interval | |
|  |  |  |  |  |  | Lower Bound | Upper Bound |
| Intercept | 337,200738 | 52,582517 | 26,694 | 6,413 | ,000 | 229,252419 | 445,149056 |
| [tidspunktR=1,00] | -132,778718 | 88,992288 | 25,146 | -1,492 | ,148 | -316,007720 | 50,450283 |
| [tidspunktR=2,00] | -53,295223 | 54,489569 | 24,371 | -,978 | ,338 | -165,665637 | 59,075191 |
| [tidspunktR=3,00] | 0^b^ | 0 | . | . | . | . | . |
| trt | 23,550833 | 78,736915 | 31,980 | ,299 | ,767 | -136,834850 | 183,936517 |
| post_trt | 3,483676 | 108,770003 | 27,751 | ,032 | ,975 | -219,411765 | 226,379117 |
| [Diagnosis=1] | 23,897180 | 61,774959 | 26,318 | ,387 | ,702 | -103,008411 | 150,802771 |
| [Diagnosis=2] | 0^b^ | 0 | . | . | . | . | . |
| [Diagnosis=1] * trt | 39,152871 | 59,615136 | 45,420 | ,657 | ,515 | -80,887543 | 159,193286 |
| [Diagnosis=2] * trt | 0^b^ | 0 | . | . | . | . | . |
| [Diagnosis=1] * post_trt | 87,961992 | 63,701787 | 22,757 | 1,381 | ,181 | -43,893076 | 219,817060 |
| [Diagnosis=2] * post_trt | 0^b^ | 0 | . | . | . | . | . |
| a. Dependent Variable: AST3. | | | | | | | |
| b. This parameter is set to zero because it is redundant. | | | | | | | |

# IED1

## ITT

| **Estimates of Fixed Effects^a^** | | | | | | | |
| --- | --- | --- | --- | --- | --- | --- | --- |
| Parameter | Estimate | Std. Error | df | t | Sig. | 95% Confidence Interval | |
|  |  |  |  |  |  | Lower Bound | Upper Bound |
| Intercept | 30,518073 | 4,368409 | 70,815 | 6,986 | ,000 | 21,807318 | 39,228827 |
| [tidspunktR=1,00] | -8,664101 | 7,664045 | 73,359 | -1,130 | ,262 | -23,937260 | 6,609058 |
| [tidspunktR=2,00] | -5,296118 | 4,921433 | 62,187 | -1,076 | ,286 | -15,133333 | 4,541098 |
| [tidspunktR=3,00] | 0^b^ | 0 | . | . | . | . | . |
| trt | 1,255746 | 5,022001 | 46,279 | ,250 | ,804 | -8,851372 | 11,362864 |
| post_trt | -,052941 | 8,335838 | 48,118 | -,006 | ,995 | -16,812208 | 16,706326 |
| a. Dependent Variable: IED1. | | | | | | | |
| b. This parameter is set to zero because it is redundant. | | | | | | | |

Diagnose

| **Estimates of Fixed Effects^a^** | | | | | | | |
| --- | --- | --- | --- | --- | --- | --- | --- |
| Parameter | Estimate | Std. Error | df | t | Sig. | 95% Confidence Interval | |
|  |  |  |  |  |  | Lower Bound | Upper Bound |
| Intercept | 28,122290 | 6,055777 | 87,827 | 4,644 | ,000 | 16,087375 | 40,157205 |
| [tidspunktR=1,00] | -9,220610 | 7,743161 | 70,116 | -1,191 | ,238 | -24,663408 | 6,222187 |
| [tidspunktR=2,00] | -5,671971 | 4,951672 | 58,962 | -1,145 | ,257 | -15,580379 | 4,236436 |
| [tidspunktR=3,00] | 0^b^ | 0 | . | . | . | . | . |
| trt | ,835600 | 7,119706 | 78,044 | ,117 | ,907 | -13,338520 | 15,009721 |
| post_trt | 4,307671 | 10,487445 | 72,651 | ,411 | ,682 | -16,595469 | 25,210812 |
| [Diagnosis=1] | 3,822483 | 6,706487 | 63,523 | ,570 | ,571 | -9,577203 | 17,222168 |
| [Diagnosis=2] | 0^b^ | 0 | . | . | . | . | . |
| [Diagnosis=1] * trt | ,608465 | 6,968324 | 73,332 | ,087 | ,931 | -13,278326 | 14,495255 |
| [Diagnosis=2] * trt | 0^b^ | 0 | . | . | . | . | . |
| [Diagnosis=1] * post_trt | -5,471994 | 8,697921 | 44,880 | -,629 | ,532 | -22,991797 | 12,047810 |
| [Diagnosis=2] * post_trt | 0^b^ | 0 | . | . | . | . | . |
| a. Dependent Variable: IED1. | | | | | | | |
| b. This parameter is set to zero because it is redundant. | | | | | | | |

## PP (80 % adherence)

| **Estimates of Fixed Effects^a^** | | | | | | | |
| --- | --- | --- | --- | --- | --- | --- | --- |
| Parameter | Estimate | Std. Error | df | t | Sig. | 95% Confidence Interval | |
|  |  |  |  |  |  | Lower Bound | Upper Bound |
| Intercept | 30,782254 | 8,719025 | 16,004 | 3,530 | ,003 | 12,299098 | 49,265410 |
| [tidspunktR=1,00] | -20,686790 | 16,751504 | 21,536 | -1,235 | ,230 | -55,470719 | 14,097140 |
| [tidspunktR=2,00] | -13,713885 | 12,145488 | 20,645 | -1,129 | ,272 | -38,998294 | 11,570525 |
| [tidspunktR=3,00] | 0^b^ | 0 | . | . | . | . | . |
| trt | 13,045103 | 10,218677 | 13,737 | 1,277 | ,223 | -8,911243 | 35,001448 |
| post_trt | 13,667586 | 16,582034 | 13,990 | ,824 | ,424 | -21,899728 | 49,234901 |
| a. Dependent Variable: IED1. | | | | | | | |
| b. This parameter is set to zero because it is redundant. | | | | | | | |

| **Estimates of Fixed Effects^a^** | | | | | | | |
| --- | --- | --- | --- | --- | --- | --- | --- |
| Parameter | Estimate | Std. Error | df | t | Sig. | 95% Confidence Interval | |
|  |  |  |  |  |  | Lower Bound | Upper Bound |
| Intercept | 32,581187 | 11,134976 | 21,977 | 2,926 | ,008 | 9,487246 | 55,675129 |
| [tidspunktR=1,00] | -18,690417 | 16,393356 | 21,093 | -1,140 | ,267 | -52,773143 | 15,392308 |
| [tidspunktR=2,00] | -12,037857 | 11,284731 | 18,145 | -1,067 | ,300 | -35,732604 | 11,656890 |
| [tidspunktR=3,00] | 0^b^ | 0 | . | . | . | . | . |
| trt | ,035521 | 12,396958 | 21,196 | ,003 | ,998 | -25,730880 | 25,801922 |
| post_trt | 12,294686 | 18,411480 | 20,234 | ,668 | ,512 | -26,082576 | 50,671948 |
| [Diagnosis=1] | -4,047550 | 11,582952 | 17,650 | -,349 | ,731 | -28,417085 | 20,321986 |
| [Diagnosis=2] | 0^b^ | 0 | . | . | . | . | . |
| [Diagnosis=1] * trt | 17,386725 | 12,612610 | 21,843 | 1,379 | ,182 | -8,781166 | 43,554615 |
| [Diagnosis=2] * trt | 0^b^ | 0 | . | . | . | . | . |
| [Diagnosis=1] * post_trt | ,170418 | 14,535550 | 17,183 | ,012 | ,991 | -30,472010 | 30,812845 |
| [Diagnosis=2] * post_trt | 0^b^ | 0 | . | . | . | . | . |
| a. Dependent Variable: IED1. | | | | | | | |
| b. This parameter is set to zero because it is redundant. | | | | | | | |

## PP (60 % adherence)

| **Estimates of Fixed Effects^a^** | | | | | | | |
| --- | --- | --- | --- | --- | --- | --- | --- |
| Parameter | Estimate | Std. Error | df | t | Sig. | 95% Confidence Interval | |
|  |  |  |  |  |  | Lower Bound | Upper Bound |
| Intercept | 28,584277 | 5,836379 | 26,144 | 4,898 | ,000 | 16,590651 | 40,577904 |
| [tidspunktR=1,00] | -6,168992 | 12,271627 | 31,241 | -,503 | ,619 | -31,189324 | 18,851341 |
| [tidspunktR=2,00] | -4,173528 | 8,588470 | 33,492 | -,486 | ,630 | -21,637153 | 13,290098 |
| [tidspunktR=3,00] | 0^b^ | 0 | . | . | . | . | . |
| trt | 2,970420 | 8,176362 | 22,171 | ,363 | ,720 | -13,978727 | 19,919568 |
| post_trt | -1,315788 | 12,549742 | 21,399 | -,105 | ,917 | -27,384793 | 24,753217 |
| a. Dependent Variable: IED1. | | | | | | | |
| b. This parameter is set to zero because it is redundant. | | | | | | | |

| **Estimates of Fixed Effects^a^** | | | | | | | |
| --- | --- | --- | --- | --- | --- | --- | --- |
| Parameter | Estimate | Std. Error | df | t | Sig. | 95% Confidence Interval | |
|  |  |  |  |  |  | Lower Bound | Upper Bound |
| Intercept | 28,400501 | 8,954304 | 35,220 | 3,172 | ,003 | 10,226352 | 46,574651 |
| [tidspunktR=1,00] | -5,962100 | 12,668047 | 28,340 | -,471 | ,641 | -31,897393 | 19,973193 |
| [tidspunktR=2,00] | -4,028368 | 8,660510 | 27,393 | -,465 | ,646 | -21,786348 | 13,729613 |
| [tidspunktR=3,00] | 0^b^ | 0 | . | . | . | . | . |
| trt | 2,389909 | 11,349010 | 35,059 | ,211 | ,834 | -20,648428 | 25,428246 |
| post_trt | 3,835881 | 15,431566 | 32,183 | ,249 | ,805 | -27,590165 | 35,261927 |
| [Diagnosis=1] | ,218501 | 9,558985 | 30,190 | ,023 | ,982 | -19,298395 | 19,735397 |
| [Diagnosis=2] | 0^b^ | 0 | . | . | . | . | . |
| [Diagnosis=1] * trt | ,408932 | 11,003164 | 36,650 | ,037 | ,971 | -21,892775 | 22,710639 |
| [Diagnosis=2] * trt | 0^b^ | 0 | . | . | . | . | . |
| [Diagnosis=1] * post_trt | -7,456075 | 12,231648 | 27,010 | -,610 | ,547 | -32,552906 | 17,640756 |
| [Diagnosis=2] * post_trt | 0^b^ | 0 | . | . | . | . | . |
| a. Dependent Variable: IED1. | | | | | | | |
| b. This parameter is set to zero because it is redundant. | | | | | | | |

# PAL1

## ITT

| **Estimates of Fixed Effects^a^** | | | | | | | |
| --- | --- | --- | --- | --- | --- | --- | --- |
| Parameter | Estimate | Std. Error | df | t | Sig. | 95% Confidence Interval | |
|  |  |  |  |  |  | Lower Bound | Upper Bound |
| Intercept | 21,826900 | 1,799104 | 68,224 | 12,132 | ,000 | 18,237057 | 25,416743 |
| [tidspunktR=1,00] | -11,221917 | 8,392869 | 44,173 | -1,337 | ,188 | -28,134761 | 5,690928 |
| [tidspunktR=2,00] | -4,168467 | 4,284379 | 42,712 | -,973 | ,336 | -12,810425 | 4,473492 |
| [tidspunktR=3,00] | 0^b^ | 0 | . | . | . | . | . |
| trt | 5,485201 | 5,943056 | 44,698 | ,923 | ,361 | -6,486958 | 17,457360 |
| post_trt | 7,677613 | 10,168176 | 41,300 | ,755 | ,454 | -12,852911 | 28,208136 |
| a. Dependent Variable: PAL1. | | | | | | | |
| b. This parameter is set to zero because it is redundant. | | | | | | | |

Diagnose

| **Estimates of Fixed Effects^a^** | | | | | | | |
| --- | --- | --- | --- | --- | --- | --- | --- |
| Parameter | Estimate | Std. Error | df | t | Sig. | 95% Confidence Interval | |
|  |  |  |  |  |  | Lower Bound | Upper Bound |
| Intercept | 23,584910 | 2,890830 | 65,124 | 8,159 | ,000 | 17,811734 | 29,358086 |
| [tidspunktR=1,00] | -10,605860 | 8,461742 | 43,891 | -1,253 | ,217 | -27,660576 | 6,448856 |
| [tidspunktR=2,00] | -3,987063 | 4,321963 | 41,813 | -,923 | ,362 | -12,710293 | 4,736166 |
| [tidspunktR=3,00] | 0^b^ | 0 | . | . | . | . | . |
| trt | 8,339309 | 6,821327 | 62,694 | 1,223 | ,226 | -5,293326 | 21,971945 |
| post_trt | 7,912839 | 10,859337 | 48,518 | ,729 | ,470 | -13,915298 | 29,740976 |
| [Diagnosis=1] | -2,804269 | 3,662285 | 63,885 | -,766 | ,447 | -10,120779 | 4,512241 |
| [Diagnosis=2] | 0^b^ | 0 | . | . | . | . | . |
| [Diagnosis=1] * trt | -4,083427 | 4,626258 | 62,739 | -,883 | ,381 | -13,329019 | 5,162164 |
| [Diagnosis=2] * trt | 0^b^ | 0 | . | . | . | . | . |
| [Diagnosis=1] * post_trt | -1,157115 | 5,565096 | 41,969 | -,208 | ,836 | -12,388180 | 10,073950 |
| [Diagnosis=2] * post_trt | 0^b^ | 0 | . | . | . | . | . |
| a. Dependent Variable: PAL1. | | | | | | | |
| b. This parameter is set to zero because it is redundant. | | | | | | | |

## PP (80 % adherence)

| **Estimates of Fixed Effects^a^** | | | | | | | |
| --- | --- | --- | --- | --- | --- | --- | --- |
| Parameter | Estimate | Std. Error | df | t | Sig. | 95% Confidence Interval | |
|  |  |  |  |  |  | Lower Bound | Upper Bound |
| Intercept | 26,355393 | 4,082207 | 16,777 | 6,456 | ,000 | 17,733978 | 34,976809 |
| [tidspunktR=1,00] | -15,238959 | 10,881633 | 17,130 | -1,400 | ,179 | -38,183927 | 7,706009 |
| [tidspunktR=2,00] | -11,657584 | 5,366130 | 17,745 | -2,172 | ,044 | -22,943039 | -,372130 |
| [tidspunktR=3,00] | 0^b^ | 0 | . | . | . | . | . |
| trt | 5,633821 | 5,890325 | 16,031 | ,956 | ,353 | -6,851127 | 18,118769 |
| post_trt | 8,341771 | 12,500041 | 16,107 | ,667 | ,514 | -18,142864 | 34,826406 |
| a. Dependent Variable: PAL1. | | | | | | | |
| b. This parameter is set to zero because it is redundant. | | | | | | | |

| **Estimates of Fixed Effects^a^** | | | | | | | |
| --- | --- | --- | --- | --- | --- | --- | --- |
| Parameter | Estimate | Std. Error | df | t | Sig. | 95% Confidence Interval | |
|  |  |  |  |  |  | Lower Bound | Upper Bound |
| Intercept | 32,668215 | 5,438394 | 18,698 | 6,007 | ,000 | 21,273067 | 44,063363 |
| [tidspunktR=1,00] | -14,787407 | 11,375876 | 16,056 | -1,300 | ,212 | -38,896321 | 9,321507 |
| [tidspunktR=2,00] | -11,237352 | 5,643109 | 15,881 | -1,991 | ,064 | -23,207504 | ,732800 |
| [tidspunktR=3,00] | 0^b^ | 0 | . | . | . | . | . |
| trt | 2,132460 | 6,962747 | 19,809 | ,306 | ,763 | -12,400573 | 16,665493 |
| post_trt | 4,557969 | 13,612165 | 16,547 | ,335 | ,742 | -24,221234 | 33,337171 |
| [Diagnosis=1] | -10,773018 | 6,164900 | 13,378 | -1,747 | ,103 | -24,053279 | 2,507243 |
| [Diagnosis=2] | 0^b^ | 0 | . | . | . | . | . |
| [Diagnosis=1] * trt | 5,293068 | 5,311861 | 24,785 | ,996 | ,329 | -5,651737 | 16,237873 |
| [Diagnosis=2] * trt | 0^b^ | 0 | . | . | . | . | . |
| [Diagnosis=1] * post_trt | 5,997999 | 7,183297 | 15,995 | ,835 | ,416 | -9,230264 | 21,226262 |
| [Diagnosis=2] * post_trt | 0^b^ | 0 | . | . | . | . | . |
| a. Dependent Variable: PAL1. | | | | | | | |
| b. This parameter is set to zero because it is redundant. | | | | | | | |

## PP (60 % adherence)

| **Estimates of Fixed Effects^a^** | | | | | | | |
| --- | --- | --- | --- | --- | --- | --- | --- |
| Parameter | Estimate | Std. Error | df | t | Sig. | 95% Confidence Interval | |
|  |  |  |  |  |  | Lower Bound | Upper Bound |
| Intercept | 26,488431 | 3,665902 | 20,081 | 7,226 | ,000 | 18,843475 | 34,133388 |
| [tidspunktR=1,00] | -11,423678 | 9,457290 | 19,080 | -1,208 | ,242 | -31,212431 | 8,365075 |
| [tidspunktR=2,00] | -6,434198 | 4,806184 | 13,649 | -1,339 | ,203 | -16,767337 | 3,898942 |
| [tidspunktR=3,00] | 0^b^ | 0 | . | . | . | . | . |
| trt | 5,140870 | 6,066990 | 14,224 | ,847 | ,411 | -7,852355 | 18,134095 |
| post_trt | 5,042576 | 10,895859 | 18,951 | ,463 | ,649 | -17,766694 | 27,851847 |
| a. Dependent Variable: PAL1. | | | | | | | |
| b. This parameter is set to zero because it is redundant. | | | | | | | |

| **Estimates of Fixed Effects^a^** | | | | | | | |
| --- | --- | --- | --- | --- | --- | --- | --- |
| Parameter | Estimate | Std. Error | df | t | Sig. | 95% Confidence Interval | |
|  |  |  |  |  |  | Lower Bound | Upper Bound |
| Intercept | 34,339782 | 5,158298 | 21,834 | 6,657 | ,000 | 23,637411 | 45,042153 |
| [tidspunktR=1,00] | -10,834370 | 13,941664 | 15,401 | -,777 | ,449 | -40,483043 | 18,814304 |
| [tidspunktR=2,00] | -5,854582 | 7,726671 | 14,653 | -,758 | ,461 | -22,357645 | 10,648480 |
| [tidspunktR=3,00] | 0^b^ | 0 | . | . | . | . | . |
| trt | -2,087373 | 9,970546 | 24,908 | -,209 | ,836 | -22,625947 | 18,451201 |
| post_trt | 4,855993 | 16,349709 | 16,606 | ,297 | ,770 | -29,701338 | 39,413324 |
| [Diagnosis=1] | -14,198368 | 5,937427 | 15,424 | -2,391 | ,030 | -26,823494 | -1,573241 |
| [Diagnosis=2] | 0^b^ | 0 | . | . | . | . | . |
| [Diagnosis=1] * trt | 10,589469 | 5,120203 | 20,223 | 2,068 | ,052 | -,083537 | 21,262474 |
| [Diagnosis=2] * trt | 0^b^ | 0 | . | . | . | . | . |
| [Diagnosis=1] * post_trt | 4,197626 | 5,730853 | 23,297 | ,732 | ,471 | -7,649193 | 16,044445 |
| [Diagnosis=2] * post_trt | 0^b^ | 0 | . | . | . | . | . |
| a. Dependent Variable: PAL1. | | | | | | | |
| b. This parameter is set to zero because it is redundant. | | | | | | | |

# PAL2

## ITT

| **Estimates of Fixed Effects^a^** | | | | | | | |
| --- | --- | --- | --- | --- | --- | --- | --- |
| Parameter | Estimate | Std. Error | df | t | Sig. | 95% Confidence Interval | |
|  |  |  |  |  |  | Lower Bound | Upper Bound |
| Intercept | 14,353461 | 1,305443 | 66,380 | 10,995 | ,000 | 11,747339 | 16,959583 |
| [tidspunktR=1,00] | -4,086011 | 6,452272 | 48,171 | -,633 | ,530 | -17,057986 | 8,885964 |
| [tidspunktR=2,00] | -2,661008 | 3,126662 | 44,961 | -,851 | ,399 | -8,958577 | 3,636560 |
| [tidspunktR=3,00] | 0^b^ | 0 | . | . | . | . | . |
| trt | 2,147405 | 4,290111 | 46,242 | ,501 | ,619 | -6,486924 | 10,781734 |
| post_trt | 5,102357 | 7,901599 | 44,531 | ,646 | ,522 | -10,816907 | 21,021621 |
| a. Dependent Variable: PAL2. | | | | | | | |
| b. This parameter is set to zero because it is redundant. | | | | | | | |

Diagnose

| **Estimates of Fixed Effects^a^** | | | | | | | |
| --- | --- | --- | --- | --- | --- | --- | --- |
| Parameter | Estimate | Std. Error | df | t | Sig. | 95% Confidence Interval | |
|  |  |  |  |  |  | Lower Bound | Upper Bound |
| Intercept | 17,085371 | 2,042881 | 63,601 | 8,363 | ,000 | 13,003754 | 21,166987 |
| [tidspunktR=1,00] | -3,508261 | 6,519731 | 47,989 | -,538 | ,593 | -16,617136 | 9,600613 |
| [tidspunktR=2,00] | -2,306875 | 3,164265 | 44,269 | -,729 | ,470 | -8,682937 | 4,069187 |
| [tidspunktR=3,00] | 0^b^ | 0 | . | . | . | . | . |
| trt | 2,780654 | 5,048178 | 66,085 | ,551 | ,584 | -7,298117 | 12,859425 |
| post_trt | 3,190090 | 8,580597 | 53,608 | ,372 | ,712 | -14,015854 | 20,396034 |
| [Diagnosis=1] | -4,442955 | 2,582276 | 61,650 | -1,721 | ,090 | -9,605433 | ,719522 |
| [Diagnosis=2] | 0^b^ | 0 | . | . | . | . | . |
| [Diagnosis=1] * trt | -,918688 | 3,661664 | 68,391 | -,251 | ,803 | -8,224668 | 6,387293 |
| [Diagnosis=2] * trt | 0^b^ | 0 | . | . | . | . | . |
| [Diagnosis=1] * post_trt | 2,217817 | 4,770565 | 45,812 | ,465 | ,644 | -7,385895 | 11,821529 |
| [Diagnosis=2] * post_trt | 0^b^ | 0 | . | . | . | . | . |
| a. Dependent Variable: PAL2. | | | | | | | |
| b. This parameter is set to zero because it is redundant. | | | | | | | |

## PP (80 % adherence)

| **Estimates of Fixed Effects^a^** | | | | | | | |
| --- | --- | --- | --- | --- | --- | --- | --- |
| Parameter | Estimate | Std. Error | df | t | Sig. | 95% Confidence Interval | |
|  |  |  |  |  |  | Lower Bound | Upper Bound |
| Intercept | 16,286236 | 2,905143 | 16,123 | 5,606 | ,000 | 10,131437 | 22,441035 |
| [tidspunktR=1,00] | -6,418239 | 8,490541 | 19,158 | -,756 | ,459 | -24,179219 | 11,342741 |
| [tidspunktR=2,00] | -7,011263 | 4,397627 | 20,360 | -1,594 | ,126 | -16,174176 | 2,151651 |
| [tidspunktR=3,00] | 0^b^ | 0 | . | . | . | . | . |
| trt | 1,692134 | 4,550283 | 15,961 | ,372 | ,715 | -7,955934 | 11,340202 |
| post_trt | 5,601938 | 9,392875 | 16,042 | ,596 | ,559 | -14,305870 | 25,509746 |
| a. Dependent Variable: PAL2. | | | | | | | |
| b. This parameter is set to zero because it is redundant. | | | | | | | |

| **Estimates of Fixed Effects^a^** | | | | | | | |
| --- | --- | --- | --- | --- | --- | --- | --- |
| Parameter | Estimate | Std. Error | df | t | Sig. | 95% Confidence Interval | |
|  |  |  |  |  |  | Lower Bound | Upper Bound |
| Intercept | 20,965391 | 3,783450 | 18,321 | 5,541 | ,000 | 13,026629 | 28,904153 |
| [tidspunktR=1,00] | -5,981418 | 8,841750 | 17,748 | -,676 | ,507 | -24,576195 | 12,613360 |
| [tidspunktR=2,00] | -6,647111 | 4,459983 | 19,457 | -1,490 | ,152 | -15,967140 | 2,672918 |
| [tidspunktR=3,00] | 0^b^ | 0 | . | . | . | . | . |
| trt | -,525076 | 5,433685 | 22,059 | -,097 | ,924 | -11,792104 | 10,741952 |
| post_trt | -,031586 | 10,462959 | 17,706 | -,003 | ,998 | -22,039625 | 21,976454 |
| [Diagnosis=1] | -7,835709 | 4,332909 | 13,215 | -1,808 | ,093 | -17,180962 | 1,509544 |
| [Diagnosis=2] | 0^b^ | 0 | . | . | . | . | . |
| [Diagnosis=1] * trt | 3,405746 | 4,523610 | 24,568 | ,753 | ,459 | -5,919114 | 12,730606 |
| [Diagnosis=2] * trt | 0^b^ | 0 | . | . | . | . | . |
| [Diagnosis=1] * post_trt | 8,769682 | 6,105594 | 17,229 | 1,436 | ,169 | -4,098943 | 21,638307 |
| [Diagnosis=2] * post_trt | 0^b^ | 0 | . | . | . | . | . |
| a. Dependent Variable: PAL2. | | | | | | | |
| b. This parameter is set to zero because it is redundant. | | | | | | | |

## PP (60 % adherence)

| **Estimates of Fixed Effects^a^** | | | | | | | |
| --- | --- | --- | --- | --- | --- | --- | --- |
| Parameter | Estimate | Std. Error | df | t | Sig. | 95% Confidence Interval | |
|  |  |  |  |  |  | Lower Bound | Upper Bound |
| Intercept | 15,965305 | 2,594678 | 18,682 | 6,153 | ,000 | 10,528307 | 21,402303 |
| [tidspunktR=1,00] | -3,241334 | 8,719464 | 21,220 | -,372 | ,714 | -21,363033 | 14,880366 |
| [tidspunktR=2,00] | -2,977221 | 4,124185 | 16,387 | -,722 | ,481 | -11,703343 | 5,748901 |
| [tidspunktR=3,00] | 0^b^ | 0 | . | . | . | . | . |
| trt | 1,644315 | 5,148381 | 16,870 | ,319 | ,753 | -9,224203 | 12,512832 |
| post_trt | 3,775934 | 10,063937 | 20,613 | ,375 | ,711 | -17,177135 | 24,729003 |
| a. Dependent Variable: PAL2. | | | | | | | |
| b. This parameter is set to zero because it is redundant. | | | | | | | |

| **Estimates of Fixed Effects^a^** | | | | | | | |
| --- | --- | --- | --- | --- | --- | --- | --- |
| Parameter | Estimate | Std. Error | df | t | Sig. | 95% Confidence Interval | |
|  |  |  |  |  |  | Lower Bound | Upper Bound |
| Intercept | 23,027307 | 4,249410 | 15,542 | 5,419 | ,000 | 13,997335 | 32,057279 |
| [tidspunktR=1,00] | -3,760302 | 9,131706 | 18,792 | -,412 | ,685 | -22,887528 | 15,366924 |
| [tidspunktR=2,00] | -3,002989 | 4,397695 | 13,749 | -,683 | ,506 | -12,451270 | 6,445291 |
| [tidspunktR=3,00] | 0^b^ | 0 | . | . | . | . | . |
| trt | ,013199 | 6,436634 | 20,275 | ,002 | ,998 | -13,401729 | 13,428128 |
| post_trt | -1,700957 | 11,447916 | 23,428 | -,149 | ,883 | -25,358847 | 21,956933 |
| [Diagnosis=1] | -10,349728 | 5,071254 | 16,046 | -2,041 | ,058 | -21,097805 | ,398349 |
| [Diagnosis=2] | 0^b^ | 0 | . | . | . | . | . |
| [Diagnosis=1] * trt | 3,108781 | 4,800100 | 37,369 | ,648 | ,521 | -6,613906 | 12,831468 |
| [Diagnosis=2] * trt | 0^b^ | 0 | . | . | . | . | . |
| [Diagnosis=1] * post_trt | 9,063687 | 6,496258 | 24,302 | 1,395 | ,176 | -4,335123 | 22,462496 |
| [Diagnosis=2] * post_trt | 0^b^ | 0 | . | . | . | . | . |
| a. Dependent Variable: PAL2. | | | | | | | |
| b. This parameter is set to zero because it is redundant. | | | | | | | |

# SST2

## ITT

| **Estimates of Fixed Effects^a^** | | | | | | | |
| --- | --- | --- | --- | --- | --- | --- | --- |
| Parameter | Estimate | Std. Error | df | t | Sig. | 95% Confidence Interval | |
|  |  |  |  |  |  | Lower Bound | Upper Bound |
| Intercept | 212,516197 | 6,603723 | 67,682 | 32,181 | ,000 | 199,337551 | 225,694844 |
| [tidspunktR=1,00] | -53,787332 | 29,734512 | 55,201 | -1,809 | ,076 | -113,371747 | 5,797084 |
| [tidspunktR=2,00] | -32,005862 | 14,011845 | 58,779 | -2,284 | ,026 | -60,045708 | -3,966016 |
| [tidspunktR=3,00] | 0^b^ | 0 | . | . | . | . | . |
| trt | 33,259829 | 18,641074 | 50,892 | 1,784 | ,080 | -4,165623 | 70,685281 |
| post_trt | 42,285694 | 36,842158 | 50,690 | 1,148 | ,256 | -31,689020 | 116,260408 |
| a. Dependent Variable: SST2. | | | | | | | |
| b. This parameter is set to zero because it is redundant. | | | | | | | |

Diagnose

| **Estimates of Fixed Effects^a^** | | | | | | | |
| --- | --- | --- | --- | --- | --- | --- | --- |
| Parameter | Estimate | Std. Error | df | t | Sig. | 95% Confidence Interval | |
|  |  |  |  |  |  | Lower Bound | Upper Bound |
| Intercept | 200,437289 | 10,459028 | 71,725 | 19,164 | ,000 | 179,586233 | 221,288344 |
| [tidspunktR=1,00] | -54,914976 | 29,978342 | 54,308 | -1,832 | ,072 | -115,010140 | 5,180189 |
| [tidspunktR=2,00] | -33,432638 | 14,170992 | 57,575 | -2,359 | ,022 | -61,803424 | -5,061853 |
| [tidspunktR=3,00] | 0^b^ | 0 | . | . | . | . | . |
| trt | 34,103173 | 23,091482 | 70,490 | 1,477 | ,144 | -11,945713 | 80,152059 |
| post_trt | 74,419131 | 40,774081 | 60,694 | 1,825 | ,073 | -7,121998 | 155,960260 |
| [Diagnosis=1] | 19,127712 | 12,969123 | 71,601 | 1,475 | ,145 | -6,728227 | 44,983650 |
| [Diagnosis=2] | 0^b^ | 0 | . | . | . | . | . |
| [Diagnosis=1] * trt | -1,310588 | 18,700561 | 77,338 | -,070 | ,944 | -38,545563 | 35,924387 |
| [Diagnosis=2] * trt | 0^b^ | 0 | . | . | . | . | . |
| [Diagnosis=1] * post_trt | -42,917500 | 25,327789 | 49,202 | -1,694 | ,096 | -93,810301 | 7,975302 |
| [Diagnosis=2] * post_trt | 0^b^ | 0 | . | . | . | . | . |
| a. Dependent Variable: SST2. | | | | | | | |
| b. This parameter is set to zero because it is redundant. | | | | | | | |

## PP (80 % adherence)

| **Estimates of Fixed Effects^a^** | | | | | | | |
| --- | --- | --- | --- | --- | --- | --- | --- |
| Parameter | Estimate | Std. Error | df | t | Sig. | 95% Confidence Interval | |
|  |  |  |  |  |  | Lower Bound | Upper Bound |
| Intercept | 206,999708 | 13,101636 | 16,452 | 15,800 | ,000 | 179,287405 | 234,712010 |
| [tidspunktR=1,00] | -59,889416 | 82,863139 | 17,129 | -,723 | ,480 | -234,615222 | 114,836390 |
| [tidspunktR=2,00] | -30,267492 | 38,535534 | 19,083 | -,785 | ,442 | -110,899665 | 50,364681 |
| [tidspunktR=3,00] | 0^b^ | 0 | . | . | . | . | . |
| trt | 30,769280 | 45,652518 | 17,026 | ,674 | ,509 | -65,537846 | 127,076406 |
| post_trt | 64,099922 | 95,154702 | 16,315 | ,674 | ,510 | -137,302937 | 265,502781 |
| a. Dependent Variable: SST2. | | | | | | | |
| b. This parameter is set to zero because it is redundant. | | | | | | | |

| **Estimates of Fixed Effects^a^** | | | | | | | |
| --- | --- | --- | --- | --- | --- | --- | --- |
| Parameter | Estimate | Std. Error | df | t | Sig. | 95% Confidence Interval | |
|  |  |  |  |  |  | Lower Bound | Upper Bound |
| Intercept | 215,387376 | 21,572712 | 17,395 | 9,984 | ,000 | 169,951446 | 260,823306 |
| [tidspunktR=1,00] | -55,555461 | 82,266071 | 16,982 | -,675 | ,509 | -229,135579 | 118,024658 |
| [tidspunktR=2,00] | -29,499869 | 38,815231 | 18,472 | -,760 | ,457 | -110,898561 | 51,898823 |
| [tidspunktR=3,00] | 0^b^ | 0 | . | . | . | . | . |
| trt | 29,106403 | 47,652742 | 18,452 | ,611 | ,549 | -70,832955 | 129,045760 |
| post_trt | 85,487644 | 95,407950 | 16,928 | ,896 | ,383 | -115,871226 | 286,846514 |
| [Diagnosis=1] | -13,262406 | 26,759472 | 16,681 | -,496 | ,627 | -69,802249 | 43,277436 |
| [Diagnosis=2] | 0^b^ | 0 | . | . | . | . | . |
| [Diagnosis=1] * trt | -4,053642 | 23,955345 | 18,946 | -,169 | ,867 | -54,202360 | 46,095077 |
| [Diagnosis=2] * trt | 0^b^ | 0 | . | . | . | . | . |
| [Diagnosis=1] * post_trt | -44,794798 | 31,695010 | 16,223 | -1,413 | ,176 | -111,910202 | 22,320606 |
| [Diagnosis=2] * post_trt | 0^b^ | 0 | . | . | . | . | . |
| a. Dependent Variable: SST2. | | | | | | | |
| b. This parameter is set to zero because it is redundant. | | | | | | | |

## PP (60 % adherence)

| **Estimates of Fixed Effects^a^** | | | | | | | |
| --- | --- | --- | --- | --- | --- | --- | --- |
| Parameter | Estimate | Std. Error | df | t | Sig. | 95% Confidence Interval | |
|  |  |  |  |  |  | Lower Bound | Upper Bound |
| Intercept | 213,711646 | 10,046950 | 26,065 | 21,271 | ,000 | 193,062343 | 234,360949 |
| [tidspunktR=1,00] | -76,672991 | 57,353301 | 26,527 | -1,337 | ,193 | -194,450545 | 41,104562 |
| [tidspunktR=2,00] | -36,227435 | 28,455091 | 30,963 | -1,273 | ,212 | -94,264790 | 21,809920 |
| [tidspunktR=3,00] | 0^b^ | 0 | . | . | . | . | . |
| trt | 40,599885 | 33,271362 | 26,329 | 1,220 | ,233 | -27,748831 | 108,948602 |
| post_trt | 69,721525 | 65,867341 | 24,872 | 1,059 | ,300 | -65,970079 | 205,413128 |
| a. Dependent Variable: SST2. | | | | | | | |
| b. This parameter is set to zero because it is redundant. | | | | | | | |

| **Estimates of Fixed Effects^a^** | | | | | | | |
| --- | --- | --- | --- | --- | --- | --- | --- |
| Parameter | Estimate | Std. Error | df | t | Sig. | 95% Confidence Interval | |
|  |  |  |  |  |  | Lower Bound | Upper Bound |
| Intercept | 217,407624 | 18,926369 | 29,022 | 11,487 | ,000 | 178,700120 | 256,115128 |
| [tidspunktR=1,00] | -75,928819 | 57,182329 | 26,179 | -1,328 | ,196 | -193,429668 | 41,572029 |
| [tidspunktR=2,00] | -36,749436 | 28,771965 | 30,070 | -1,277 | ,211 | -95,503894 | 22,005021 |
| [tidspunktR=3,00] | 0^b^ | 0 | . | . | . | . | . |
| trt | 47,402279 | 38,444044 | 33,407 | 1,233 | ,226 | -30,776539 | 125,581098 |
| post_trt | 111,220975 | 69,216238 | 27,956 | 1,607 | ,119 | -30,572204 | 253,014154 |
| [Diagnosis=1] | -4,996682 | 21,628261 | 28,049 | -,231 | ,819 | -49,296703 | 39,303339 |
| [Diagnosis=2] | 0^b^ | 0 | . | . | . | . | . |
| [Diagnosis=1] * trt | -11,919952 | 26,177326 | 36,435 | -,455 | ,652 | -64,988039 | 41,148135 |
| [Diagnosis=2] * trt | 0^b^ | 0 | . | . | . | . | . |
| [Diagnosis=1] * post_trt | -60,370048 | 32,595385 | 27,744 | -1,852 | ,075 | -127,166476 | 6,426379 |
| [Diagnosis=2] * post_trt | 0^b^ | 0 | . | . | . | . | . |
| a. Dependent Variable: SST2. | | | | | | | |
| b. This parameter is set to zero because it is redundant. | | | | | | | |

# SST6

## ITT

| **Estimates of Fixed Effects^a^** | | | | | | | |
| --- | --- | --- | --- | --- | --- | --- | --- |
| Parameter | Estimate | Std. Error | df | t | Sig. | 95% Confidence Interval | |
|  |  |  |  |  |  | Lower Bound | Upper Bound |
| Intercept | ,539888 | ,009425 | 67,882 | 57,284 | ,000 | ,521081 | ,558696 |
| [tidspunktR=1,00] | -,011458 | ,036946 | 57,338 | -,310 | ,758 | -,085431 | ,062515 |
| [tidspunktR=2,00] | -,015051 | ,019424 | 56,169 | -,775 | ,442 | -,053959 | ,023857 |
| [tidspunktR=3,00] | 0^b^ | 0 | . | . | . | . | . |
| trt | ,009324 | ,025112 | 46,885 | ,371 | ,712 | -,041197 | ,059845 |
| post_trt | -,011017 | ,044616 | 50,137 | -,247 | ,806 | -,100625 | ,078590 |
| a. Dependent Variable: SST6. | | | | | | | |
| b. This parameter is set to zero because it is redundant. | | | | | | | |

Diagnose

| **Estimates of Fixed Effects^a^** | | | | | | | |
| --- | --- | --- | --- | --- | --- | --- | --- |
| Parameter | Estimate | Std. Error | df | t | Sig. | 95% Confidence Interval | |
|  |  |  |  |  |  | Lower Bound | Upper Bound |
| Intercept | ,539253 | ,015031 | 73,756 | 35,875 | ,000 | ,509301 | ,569205 |
| [tidspunktR=1,00] | -,012546 | ,037430 | 56,534 | -,335 | ,739 | -,087512 | ,062420 |
| [tidspunktR=2,00] | -,015166 | ,019636 | 55,515 | -,772 | ,443 | -,054509 | ,024178 |
| [tidspunktR=3,00] | 0^b^ | 0 | . | . | . | . | . |
| trt | ,002580 | ,033010 | 67,361 | ,078 | ,938 | -,063301 | ,068461 |
| post_trt | -,013679 | ,052689 | 60,791 | -,260 | ,796 | -,119045 | ,091687 |
| [Diagnosis=1] | ,001056 | ,018459 | 72,587 | ,057 | ,955 | -,035737 | ,037848 |
| [Diagnosis=2] | 0^b^ | 0 | . | . | . | . | . |
| [Diagnosis=1] * trt | ,009622 | ,029690 | 87,471 | ,324 | ,747 | -,049384 | ,068629 |
| [Diagnosis=2] * trt | 0^b^ | 0 | . | . | . | . | . |
| [Diagnosis=1] * post_trt | ,005393 | ,038971 | 43,219 | ,138 | ,891 | -,073188 | ,083974 |
| [Diagnosis=2] * post_trt | 0^b^ | 0 | . | . | . | . | . |
| a. Dependent Variable: SST6. | | | | | | | |
| b. This parameter is set to zero because it is redundant. | | | | | | | |

## PP (80 % adherence)

| **Estimates of Fixed Effects^a^** | | | | | | | |
| --- | --- | --- | --- | --- | --- | --- | --- |
| Parameter | Estimate | Std. Error | df | t | Sig. | 95% Confidence Interval | |
|  |  |  |  |  |  | Lower Bound | Upper Bound |
| Intercept | ,535769 | ,016628 | 16,972 | 32,221 | ,000 | ,500683 | ,570855 |
| [tidspunktR=1,00] | -,030943 | ,079680 | 18,667 | -,388 | ,702 | -,197917 | ,136031 |
| [tidspunktR=2,00] | -,036030 | ,043643 | 18,766 | -,826 | ,419 | -,127452 | ,055392 |
| [tidspunktR=3,00] | 0^b^ | 0 | . | . | . | . | . |
| trt | ,056988 | ,050143 | 16,326 | 1,137 | ,272 | -,049137 | ,163114 |
| post_trt | ,033017 | ,090008 | 17,623 | ,367 | ,718 | -,156373 | ,222407 |
| a. Dependent Variable: SST6. | | | | | | | |
| b. This parameter is set to zero because it is redundant. | | | | | | | |

| **Estimates of Fixed Effects^a^** | | | | | | | |
| --- | --- | --- | --- | --- | --- | --- | --- |
| Parameter | Estimate | Std. Error | df | t | Sig. | 95% Confidence Interval | |
|  |  |  |  |  |  | Lower Bound | Upper Bound |
| Intercept | ,545258 | ,027150 | 17,131 | 20,083 | ,000 | ,488009 | ,602506 |
| [tidspunktR=1,00] | -,039007 | ,080821 | 17,044 | -,483 | ,635 | -,209491 | ,131477 |
| [tidspunktR=2,00] | -,037338 | ,044480 | 17,559 | -,839 | ,413 | -,130955 | ,056279 |
| [tidspunktR=3,00] | 0^b^ | 0 | . | . | . | . | . |
| trt | ,018244 | ,055191 | 19,797 | ,331 | ,744 | -,096959 | ,133447 |
| post_trt | -,013702 | ,093743 | 17,675 | -,146 | ,885 | -,210909 | ,183504 |
| [Diagnosis=1] | -,015154 | ,033648 | 16,056 | -,450 | ,658 | -,086464 | ,056156 |
| [Diagnosis=2] | 0^b^ | 0 | . | . | . | . | . |
| [Diagnosis=1] * trt | ,065430 | ,042251 | 26,074 | 1,549 | ,134 | -,021407 | ,152267 |
| [Diagnosis=2] * trt | 0^b^ | 0 | . | . | . | . | . |
| [Diagnosis=1] * post_trt | ,096060 | ,050150 | 15,558 | 1,915 | ,074 | -,010500 | ,202620 |
| [Diagnosis=2] * post_trt | 0^b^ | 0 | . | . | . | . | . |
| a. Dependent Variable: SST6. | | | | | | | |
| b. This parameter is set to zero because it is redundant. | | | | | | | |

## PP (60 % adherence)

| **Estimates of Fixed Effects^a^** | | | | | | | |
| --- | --- | --- | --- | --- | --- | --- | --- |
| Parameter | Estimate | Std. Error | df | t | Sig. | 95% Confidence Interval | |
|  |  |  |  |  |  | Lower Bound | Upper Bound |
| Intercept | ,553099 | ,015779 | 26,559 | 35,053 | ,000 | ,520698 | ,585499 |
| [tidspunktR=1,00] | -,014525 | ,071913 | 27,956 | -,202 | ,841 | -,161844 | ,132793 |
| [tidspunktR=2,00] | -,027212 | ,041203 | 27,453 | -,660 | ,514 | -,111688 | ,057264 |
| [tidspunktR=3,00] | 0^b^ | 0 | . | . | . | . | . |
| trt | ,019601 | ,048406 | 24,796 | ,405 | ,689 | -,080135 | ,119336 |
| post_trt | -,018334 | ,080743 | 25,898 | -,227 | ,822 | -,184337 | ,147668 |
| a. Dependent Variable: SST6. | | | | | | | |
| b. This parameter is set to zero because it is redundant. | | | | | | | |

| **Estimates of Fixed Effects^a^** | | | | | | | |
| --- | --- | --- | --- | --- | --- | --- | --- |
| Parameter | Estimate | Std. Error | df | t | Sig. | 95% Confidence Interval | |
|  |  |  |  |  |  | Lower Bound | Upper Bound |
| Intercept | ,562490 | ,030641 | 28,031 | 18,357 | ,000 | ,499727 | ,625253 |
| [tidspunktR=1,00] | -,013245 | ,072731 | 26,793 | -,182 | ,857 | -,162530 | ,136040 |
| [tidspunktR=2,00] | -,027804 | ,041300 | 27,022 | -,673 | ,507 | -,112542 | ,056933 |
| [tidspunktR=3,00] | 0^b^ | 0 | . | . | . | . | . |
| trt | -,013965 | ,057006 | 30,937 | -,245 | ,808 | -,130239 | ,102310 |
| post_trt | -,048986 | ,088312 | 28,215 | -,555 | ,583 | -,229822 | ,131850 |
| [Diagnosis=1] | -,012538 | ,035536 | 27,528 | -,353 | ,727 | -,085386 | ,060310 |
| [Diagnosis=2] | 0^b^ | 0 | . | . | . | . | . |
| [Diagnosis=1] * trt | ,046405 | ,042620 | 41,459 | 1,089 | ,283 | -,039640 | ,132449 |
| [Diagnosis=2] * trt | 0^b^ | 0 | . | . | . | . | . |
| [Diagnosis=1] * post_trt | ,041512 | ,048776 | 24,711 | ,851 | ,403 | -,059004 | ,142028 |
| [Diagnosis=2] * post_trt | 0^b^ | 0 | . | . | . | . | . |
| a. Dependent Variable: SST6. | | | | | | | |
| b. This parameter is set to zero because it is redundant. | | | | | | | |

# SST7

## ITT

| **Estimates of Fixed Effects^a^** | | | | | | | |
| --- | --- | --- | --- | --- | --- | --- | --- |
| Parameter | Estimate | Std. Error | df | t | Sig. | 95% Confidence Interval | |
|  |  |  |  |  |  | Lower Bound | Upper Bound |
| Intercept | 338,344536 | 17,364260 | 66,607 | 19,485 | ,000 | 303,681569 | 373,007502 |
| [tidspunktR=1,00] | 38,447165 | 58,384313 | 54,226 | ,659 | ,513 | -78,595169 | 155,489498 |
| [tidspunktR=2,00] | 32,965843 | 28,131448 | 53,814 | 1,172 | ,246 | -23,438778 | 89,370463 |
| [tidspunktR=3,00] | 0^b^ | 0 | . | . | . | . | . |
| trt | -49,707953 | 37,952633 | 48,958 | -1,310 | ,196 | -125,978268 | 26,562362 |
| post_trt | -64,779769 | 71,345743 | 49,747 | -,908 | ,368 | -208,099997 | 78,540459 |
| a. Dependent Variable: SST7. | | | | | | | |
| b. This parameter is set to zero because it is redundant. | | | | | | | |

Diagnose

| **Estimates of Fixed Effects^a^** | | | | | | | |
| --- | --- | --- | --- | --- | --- | --- | --- |
| Parameter | Estimate | Std. Error | df | t | Sig. | 95% Confidence Interval | |
|  |  |  |  |  |  | Lower Bound | Upper Bound |
| Intercept | 351,922705 | 28,343016 | 68,390 | 12,417 | ,000 | 295,370935 | 408,474476 |
| [tidspunktR=1,00] | 39,388782 | 59,057294 | 54,013 | ,667 | ,508 | -79,013294 | 157,790858 |
| [tidspunktR=2,00] | 33,738757 | 28,323389 | 53,567 | 1,191 | ,239 | -23,056742 | 90,534255 |
| [tidspunktR=3,00] | 0^b^ | 0 | . | . | . | . | . |
| trt | -51,315351 | 45,947981 | 68,359 | -1,117 | ,268 | -142,994406 | 40,363704 |
| post_trt | -89,021123 | 79,006113 | 60,808 | -1,127 | ,264 | -247,013697 | 68,971451 |
| [Diagnosis=1] | -21,506234 | 35,469920 | 68,307 | -,606 | ,546 | -92,279596 | 49,267128 |
| [Diagnosis=2] | 0^b^ | 0 | . | . | . | . | . |
| [Diagnosis=1] * trt | 2,400211 | 35,910877 | 76,135 | ,067 | ,947 | -69,120452 | 73,920873 |
| [Diagnosis=2] * trt | 0^b^ | 0 | . | . | . | . | . |
| [Diagnosis=1] * post_trt | 32,614601 | 47,484099 | 51,747 | ,687 | ,495 | -62,680315 | 127,909517 |
| [Diagnosis=2] * post_trt | 0^b^ | 0 | . | . | . | . | . |
| a. Dependent Variable: SST7. | | | | | | | |
| b. This parameter is set to zero because it is redundant. | | | | | | | |

## PP (80 % adherence)

| **Estimates of Fixed Effects^a^** | | | | | | | |
| --- | --- | --- | --- | --- | --- | --- | --- |
| Parameter | Estimate | Std. Error | df | t | Sig. | 95% Confidence Interval | |
|  |  |  |  |  |  | Lower Bound | Upper Bound |
| Intercept | 370,147128 | 35,352258 | 12,738 | 10,470 | ,000 | 293,613395 | 446,680861 |
| [tidspunktR=1,00] | 62,701306 | 146,635815 | 17,867 | ,428 | ,674 | -245,532965 | 370,935577 |
| [tidspunktR=2,00] | 65,331005 | 71,693082 | 18,701 | ,911 | ,374 | -84,886813 | 215,548823 |
| [tidspunktR=3,00] | 0^b^ | 0 | . | . | . | . | . |
| trt | -55,046201 | 85,644769 | 17,210 | -,643 | ,529 | -235,572712 | 125,480309 |
| post_trt | -99,816217 | 166,427666 | 16,790 | -,600 | ,557 | -451,283531 | 251,651097 |
| a. Dependent Variable: SST7. | | | | | | | |
| b. This parameter is set to zero because it is redundant. | | | | | | | |

| **Estimates of Fixed Effects^a^** | | | | | | | |
| --- | --- | --- | --- | --- | --- | --- | --- |
| Parameter | Estimate | Std. Error | df | t | Sig. | 95% Confidence Interval | |
|  |  |  |  |  |  | Lower Bound | Upper Bound |
| Intercept | 390,173398 | 62,642795 | 11,150 | 6,229 | ,000 | 252,522806 | 527,823989 |
| [tidspunktR=1,00] | 43,913070 | 148,041672 | 17,777 | ,297 | ,770 | -267,391254 | 355,217394 |
| [tidspunktR=2,00] | 53,483113 | 71,394421 | 18,468 | ,749 | ,463 | -96,238933 | 203,205159 |
| [tidspunktR=3,00] | 0^b^ | 0 | . | . | . | . | . |
| trt | -34,350043 | 89,066100 | 19,463 | -,386 | ,704 | -220,467642 | 151,767556 |
| post_trt | -108,887147 | 171,826542 | 17,955 | -,634 | ,534 | -469,946889 | 252,172595 |
| [Diagnosis=1] | -24,385594 | 78,824908 | 10,869 | -,309 | ,763 | -198,133951 | 149,362762 |
| [Diagnosis=2] | 0^b^ | 0 | . | . | . | . | . |
| [Diagnosis=1] * trt | -12,942207 | 45,786951 | 19,928 | -,283 | ,780 | -108,474301 | 82,589888 |
| [Diagnosis=2] * trt | 0^b^ | 0 | . | . | . | . | . |
| [Diagnosis=1] * post_trt | 40,342853 | 65,827876 | 16,269 | ,613 | ,548 | -99,018405 | 179,704111 |
| [Diagnosis=2] * post_trt | 0^b^ | 0 | . | . | . | . | . |
| a. Dependent Variable: SST7. | | | | | | | |
| b. This parameter is set to zero because it is redundant. | | | | | | | |

## PP (60 % adherence)

| **Estimates of Fixed Effects^a^** | | | | | | | |
| --- | --- | --- | --- | --- | --- | --- | --- |
| Parameter | Estimate | Std. Error | df | t | Sig. | 95% Confidence Interval | |
|  |  |  |  |  |  | Lower Bound | Upper Bound |
| Intercept | 376,337376 | 28,202648 | 24,083 | 13,344 | ,000 | 318,140577 | 434,534174 |
| [tidspunktR=1,00] | 105,319459 | 113,799558 | 27,268 | ,925 | ,363 | -128,070494 | 338,709412 |
| [tidspunktR=2,00] | 52,861180 | 58,882408 | 29,534 | ,898 | ,377 | -67,472386 | 173,194746 |
| [tidspunktR=3,00] | 0^b^ | 0 | . | . | . | . | . |
| trt | -81,404027 | 69,365057 | 25,561 | -1,174 | ,251 | -224,105198 | 61,297144 |
| post_trt | -154,454506 | 128,470772 | 24,877 | -1,202 | ,241 | -419,111155 | 110,202143 |
| a. Dependent Variable: SST7. | | | | | | | |
| b. This parameter is set to zero because it is redundant. | | | | | | | |

| **Estimates of Fixed Effects^a^** | | | | | | | |
| --- | --- | --- | --- | --- | --- | --- | --- |
| Parameter | Estimate | Std. Error | df | t | Sig. | 95% Confidence Interval | |
|  |  |  |  |  |  | Lower Bound | Upper Bound |
| Intercept | 411,555137 | 53,744131 | 26,203 | 7,658 | ,000 | 301,124199 | 521,986075 |
| [tidspunktR=1,00] | 97,451963 | 116,640478 | 26,243 | ,835 | ,411 | -142,197810 | 337,101735 |
| [tidspunktR=2,00] | 48,751187 | 59,583835 | 28,594 | ,818 | ,420 | -73,186714 | 170,689088 |
| [tidspunktR=3,00] | 0^b^ | 0 | . | . | . | . | . |
| trt | -102,731571 | 78,200033 | 32,739 | -1,314 | ,198 | -261,878905 | 56,415762 |
| post_trt | -224,229034 | 138,447018 | 27,906 | -1,620 | ,117 | -507,867874 | 59,409805 |
| [Diagnosis=1] | -47,187468 | 62,118996 | 25,182 | -,760 | ,455 | -175,077045 | 80,702110 |
| [Diagnosis=2] | 0^b^ | 0 | . | . | . | . | . |
| [Diagnosis=1] * trt | 38,328710 | 48,128622 | 33,449 | ,796 | ,431 | -59,539845 | 136,197266 |
| [Diagnosis=2] * trt | 0^b^ | 0 | . | . | . | . | . |
| [Diagnosis=1] * post_trt | 107,234121 | 61,680289 | 25,142 | 1,739 | ,094 | -19,762405 | 234,230648 |
| [Diagnosis=2] * post_trt | 0^b^ | 0 | . | . | . | . | . |
| a. Dependent Variable: SST7. | | | | | | | |
| b. This parameter is set to zero because it is redundant. | | | | | | | |

# SWM2

## ITT

| **Estimates of Fixed Effects^a^** | | | | | | | |
| --- | --- | --- | --- | --- | --- | --- | --- |
| Parameter | Estimate | Std. Error | df | t | Sig. | 95% Confidence Interval | |
|  |  |  |  |  |  | Lower Bound | Upper Bound |
| Intercept | 32,527778 | ,787960 | 71,000 | 41,281 | ,000 | 30,956630 | 34,098926 |
| [tidspunktR=1,00] | -3,194337 | 1,741951 | 52,533 | -1,834 | ,072 | -6,688974 | ,300299 |
| [tidspunktR=2,00] | -1,937601 | ,891360 | 52,721 | -2,174 | ,034 | -3,725664 | -,149537 |
| [tidspunktR=3,00] | 0^b^ | 0 | . | . | . | . | . |
| trt | 2,176080 | 1,212745 | 47,873 | 1,794 | ,079 | -,262474 | 4,614633 |
| post_trt | 3,392947 | 2,094300 | 44,154 | 1,620 | ,112 | -,827422 | 7,613317 |
| a. Dependent Variable: SWM2. | | | | | | | |
| b. This parameter is set to zero because it is redundant. | | | | | | | |

Diagnose

| **Estimates of Fixed Effects^a^** | | | | | | | |
| --- | --- | --- | --- | --- | --- | --- | --- |
| Parameter | Estimate | Std. Error | df | t | Sig. | 95% Confidence Interval | |
|  |  |  |  |  |  | Lower Bound | Upper Bound |
| Intercept | 30,139614 | 1,212721 | 73,353 | 24,853 | ,000 | 27,722860 | 32,556368 |
| [tidspunktR=1,00] | -3,495313 | 1,786184 | 49,960 | -1,957 | ,056 | -7,083041 | ,092415 |
| [tidspunktR=2,00] | -2,096691 | ,901312 | 51,085 | -2,326 | ,024 | -3,906077 | -,287305 |
| [tidspunktR=3,00] | 0^b^ | 0 | . | . | . | . | . |
| trt | 2,541148 | 1,601838 | 73,047 | 1,586 | ,117 | -,651275 | 5,733572 |
| post_trt | 2,113594 | 2,498688 | 53,828 | ,846 | ,401 | -2,896340 | 7,123528 |
| [Diagnosis=1] | 3,821062 | 1,515830 | 73,277 | 2,521 | ,014 | ,800210 | 6,841913 |
| [Diagnosis=2] | 0^b^ | 0 | . | . | . | . | . |
| [Diagnosis=1] * trt | -,322829 | 1,407420 | 85,080 | -,229 | ,819 | -3,121119 | 2,475462 |
| [Diagnosis=2] * trt | 0^b^ | 0 | . | . | . | . | . |
| [Diagnosis=1] * post_trt | 2,017226 | 1,847726 | 43,032 | 1,092 | ,281 | -1,708987 | 5,743439 |
| [Diagnosis=2] * post_trt | 0^b^ | 0 | . | . | . | . | . |
| a. Dependent Variable: SWM2. | | | | | | | |
| b. This parameter is set to zero because it is redundant. | | | | | | | |

## PP (80 % adherence)

| **Estimates of Fixed Effects^a^** | | | | | | | |
| --- | --- | --- | --- | --- | --- | --- | --- |
| Parameter | Estimate | Std. Error | df | t | Sig. | 95% Confidence Interval | |
|  |  |  |  |  |  | Lower Bound | Upper Bound |
| Intercept | 33,111111 | 1,859245 | 17,000 | 17,809 | ,000 | 29,188448 | 37,033774 |
| [tidspunktR=1,00] | -3,302716 | 2,444110 | 17,901 | -1,351 | ,193 | -8,439634 | 1,834201 |
| [tidspunktR=2,00] | -2,367263 | 1,441607 | 17,351 | -1,642 | ,119 | -5,404102 | ,669575 |
| [tidspunktR=3,00] | 0^b^ | 0 | . | . | . | . | . |
| trt | 2,579214 | 1,643034 | 14,250 | 1,570 | ,138 | -,938950 | 6,097378 |
| post_trt | 3,247801 | 2,720770 | 15,668 | 1,194 | ,250 | -2,529917 | 9,025518 |
| a. Dependent Variable: SWM2. | | | | | | | |
| b. This parameter is set to zero because it is redundant. | | | | | | | |

| **Estimates of Fixed Effects^a^** | | | | | | | |
| --- | --- | --- | --- | --- | --- | --- | --- |
| Parameter | Estimate | Std. Error | df | t | Sig. | 95% Confidence Interval | |
|  |  |  |  |  |  | Lower Bound | Upper Bound |
| Intercept | 30,546417 | 2,693422 | 17,404 | 11,341 | ,000 | 24,873834 | 36,219001 |
| [tidspunktR=1,00] | -3,606877 | 2,598365 | 15,193 | -1,388 | ,185 | -9,139046 | 1,925292 |
| [tidspunktR=2,00] | -2,371158 | 1,424693 | 13,978 | -1,664 | ,118 | -5,427268 | ,684952 |
| [tidspunktR=3,00] | 0^b^ | 0 | . | . | . | . | . |
| trt | 3,703951 | 2,094039 | 16,807 | 1,769 | ,095 | -,717955 | 8,125856 |
| post_trt | 2,084603 | 3,157539 | 16,776 | ,660 | ,518 | -4,584001 | 8,753207 |
| [Diagnosis=1] | 4,196772 | 3,315375 | 15,515 | 1,266 | ,224 | -2,849401 | 11,242944 |
| [Diagnosis=2] | 0^b^ | 0 | . | . | . | . | . |
| [Diagnosis=1] * trt | -1,723858 | 1,895029 | 25,654 | -,910 | ,371 | -5,621705 | 2,173989 |
| [Diagnosis=2] * trt | 0^b^ | 0 | . | . | . | . | . |
| [Diagnosis=1] * post_trt | 2,797182 | 2,211566 | 14,837 | 1,265 | ,225 | -1,921178 | 7,515542 |
| [Diagnosis=2] * post_trt | 0^b^ | 0 | . | . | . | . | . |
| a. Dependent Variable: SWM2. | | | | | | | |
| b. This parameter is set to zero because it is redundant. | | | | | | | |

## PP (60 % adherence)

| **Estimates of Fixed Effects^a^** | | | | | | | |
| --- | --- | --- | --- | --- | --- | --- | --- |
| Parameter | Estimate | Std. Error | df | t | Sig. | 95% Confidence Interval | |
|  |  |  |  |  |  | Lower Bound | Upper Bound |
| Intercept | 33,321429 | 1,333386 | 27,000 | 24,990 | ,000 | 30,585546 | 36,057312 |
| [tidspunktR=1,00] | -,123989 | 2,085345 | 27,776 | -,059 | ,953 | -4,397180 | 4,149202 |
| [tidspunktR=2,00] | -,741037 | 1,102054 | 23,330 | -,672 | ,508 | -3,019025 | 1,536951 |
| [tidspunktR=3,00] | 0^b^ | 0 | . | . | . | . | . |
| trt | ,152601 | 1,365660 | 22,209 | ,112 | ,912 | -2,678062 | 2,983264 |
| post_trt | -,367413 | 2,343595 | 23,755 | -,157 | ,877 | -5,206992 | 4,472166 |
| a. Dependent Variable: SWM2. | | | | | | | |
| b. This parameter is set to zero because it is redundant. | | | | | | | |

| **Estimates of Fixed Effects^a^** | | | | | | | |
| --- | --- | --- | --- | --- | --- | --- | --- |
| Parameter | Estimate | Std. Error | df | t | Sig. | 95% Confidence Interval | |
|  |  |  |  |  |  | Lower Bound | Upper Bound |
| Intercept | 29,486472 | 2,371534 | 27,172 | 12,434 | ,000 | 24,621927 | 34,351017 |
| [tidspunktR=1,00] | -,167038 | 2,149950 | 26,405 | -,078 | ,939 | -4,583024 | 4,248948 |
| [tidspunktR=2,00] | -,639212 | 1,095649 | 20,993 | -,583 | ,566 | -2,917784 | 1,639360 |
| [tidspunktR=3,00] | 0^b^ | 0 | . | . | . | . | . |
| trt | 1,088645 | 1,915529 | 30,567 | ,568 | ,574 | -2,820347 | 4,997636 |
| post_trt | -,778180 | 2,883417 | 28,226 | -,270 | ,789 | -6,682466 | 5,126106 |
| [Diagnosis=1] | 5,368939 | 2,791341 | 27,545 | 1,923 | ,065 | -,353119 | 11,090997 |
| [Diagnosis=2] | 0^b^ | 0 | . | . | . | . | . |
| [Diagnosis=1] * trt | -1,402877 | 1,706050 | 41,194 | -,822 | ,416 | -4,847823 | 2,042069 |
| [Diagnosis=2] * trt | 0^b^ | 0 | . | . | . | . | . |
| [Diagnosis=1] * post_trt | ,718236 | 2,246230 | 24,422 | ,320 | ,752 | -3,913514 | 5,349985 |
| [Diagnosis=2] * post_trt | 0^b^ | 0 | . | . | . | . | . |
| a. Dependent Variable: SWM2. | | | | | | | |
| b. This parameter is set to zero because it is redundant. | | | | | | | |

# SWM3

## ITT

| **Estimates of Fixed Effects^a^** | | | | | | | |
| --- | --- | --- | --- | --- | --- | --- | --- |
| Parameter | Estimate | Std. Error | df | t | Sig. | 95% Confidence Interval | |
|  |  |  |  |  |  | Lower Bound | Upper Bound |
| Intercept | 27,569444 | 2,199447 | 71 | 12,535 | ,000 | 23,183871 | 31,955018 |
| [tidspunktR=1,00] | -8,211262 | 4,911461 | 57,281 | -1,672 | ,100 | -18,045246 | 1,622722 |
| [tidspunktR=2,00] | -3,302249 | 2,513101 | 51,796 | -1,314 | ,195 | -8,345629 | 1,741130 |
| [tidspunktR=3,00] | 0^b^ | 0 | . | . | . | . | . |
| trt | 3,785718 | 3,432047 | 47,571 | 1,103 | ,276 | -3,116481 | 10,687918 |
| post_trt | 7,998566 | 5,849516 | 49,210 | 1,367 | ,178 | -3,755205 | 19,752337 |
| a. Dependent Variable: SWM3. | | | | | | | |
| b. This parameter is set to zero because it is redundant. | | | | | | | |

Diagnose

| **Estimates of Fixed Effects^a^** | | | | | | | |
| --- | --- | --- | --- | --- | --- | --- | --- |
| Parameter | Estimate | Std. Error | df | t | Sig. | 95% Confidence Interval | |
|  |  |  |  |  |  | Lower Bound | Upper Bound |
| Intercept | 23,237759 | 3,499275 | 72,990 | 6,641 | ,000 | 16,263697 | 30,211820 |
| [tidspunktR=1,00] | -8,671670 | 4,955145 | 56,250 | -1,750 | ,086 | -18,597043 | 1,253703 |
| [tidspunktR=2,00] | -3,530505 | 2,552612 | 50,016 | -1,383 | ,173 | -8,657534 | 1,596525 |
| [tidspunktR=3,00] | 0^b^ | 0 | . | . | . | . | . |
| trt | 2,962432 | 4,552398 | 73,809 | ,651 | ,517 | -6,108808 | 12,033672 |
| post_trt | 11,486018 | 6,845640 | 63,547 | 1,678 | ,098 | -2,191595 | 25,163632 |
| [Diagnosis=1] | 6,930697 | 4,388340 | 73,817 | 1,579 | ,119 | -1,813620 | 15,675015 |
| [Diagnosis=2] | 0^b^ | 0 | . | . | . | . | . |
| [Diagnosis=1] * trt | ,938581 | 3,973893 | 87,791 | ,236 | ,814 | -6,958958 | 8,836119 |
| [Diagnosis=2] * trt | 0^b^ | 0 | . | . | . | . | . |
| [Diagnosis=1] * post_trt | -4,339112 | 5,099887 | 49,485 | -,851 | ,399 | -14,585180 | 5,906955 |
| [Diagnosis=2] * post_trt | 0^b^ | 0 | . | . | . | . | . |
| a. Dependent Variable: SWM3. | | | | | | | |
| b. This parameter is set to zero because it is redundant. | | | | | | | |

## PP (80 % adherence)

| **Estimates of Fixed Effects^a^** | | | | | | | |
| --- | --- | --- | --- | --- | --- | --- | --- |
| Parameter | Estimate | Std. Error | df | t | Sig. | 95% Confidence Interval | |
|  |  |  |  |  |  | Lower Bound | Upper Bound |
| Intercept | 33,888889 | 4,944059 | 17,000 | 6,854 | ,000 | 23,457836 | 44,319941 |
| [tidspunktR=1,00] | -16,562863 | 7,282017 | 19,175 | -2,274 | ,035 | -31,794876 | -1,330849 |
| [tidspunktR=2,00] | -9,495608 | 3,062076 | 15,650 | -3,101 | ,007 | -15,998729 | -2,992487 |
| [tidspunktR=3,00] | 0^b^ | 0 | . | . | . | . | . |
| trt | 5,749483 | 3,689605 | 14,056 | 1,558 | ,141 | -2,160962 | 13,659929 |
| post_trt | 13,386219 | 8,055493 | 15,315 | 1,662 | ,117 | -3,752974 | 30,525413 |
| a. Dependent Variable: SWM3. | | | | | | | |
| b. This parameter is set to zero because it is redundant. | | | | | | | |

| **Estimates of Fixed Effects^a^** | | | | | | | |
| --- | --- | --- | --- | --- | --- | --- | --- |
| Parameter | Estimate | Std. Error | df | t | Sig. | 95% Confidence Interval | |
|  |  |  |  |  |  | Lower Bound | Upper Bound |
| Intercept | 23,872197 | 7,718696 | 17,645 | 3,093 | ,006 | 7,632400 | 40,111994 |
| [tidspunktR=1,00] | -17,261138 | 7,198666 | 18,056 | -2,398 | ,028 | -32,381590 | -2,140686 |
| [tidspunktR=2,00] | -9,627750 | 2,986045 | 13,374 | -3,224 | ,006 | -16,060438 | -3,195061 |
| [tidspunktR=3,00] | 0^b^ | 0 | . | . | . | . | . |
| trt | 8,707130 | 4,791590 | 15,962 | 1,817 | ,088 | -1,452539 | 18,866798 |
| post_trt | 18,866520 | 9,274329 | 18,346 | 2,034 | ,057 | -,591806 | 38,324846 |
| [Diagnosis=1] | 16,390951 | 9,626295 | 18,985 | 1,703 | ,105 | -3,758224 | 36,540125 |
| [Diagnosis=2] | 0^b^ | 0 | . | . | . | . | . |
| [Diagnosis=1] * trt | -4,474207 | 4,644852 | 20,787 | -,963 | ,346 | -14,139736 | 5,191322 |
| [Diagnosis=2] * trt | 0^b^ | 0 | . | . | . | . | . |
| [Diagnosis=1] * post_trt | -7,577672 | 8,238336 | 18,624 | -,920 | ,369 | -24,844319 | 9,688976 |
| [Diagnosis=2] * post_trt | 0^b^ | 0 | . | . | . | . | . |
| a. Dependent Variable: SWM3. | | | | | | | |
| b. This parameter is set to zero because it is redundant. | | | | | | | |

## PP (60 % adherence)

| **Estimates of Fixed Effects^a^** | | | | | | | |
| --- | --- | --- | --- | --- | --- | --- | --- |
| Parameter | Estimate | Std. Error | df | t | Sig. | 95% Confidence Interval | |
|  |  |  |  |  |  | Lower Bound | Upper Bound |
| Intercept | 30,821429 | 3,829036 | 27 | 8,049 | ,000 | 22,964895 | 38,677962 |
| [tidspunktR=1,00] | -11,425925 | 8,175968 | 26,596 | -1,398 | ,174 | -28,213571 | 5,361721 |
| [tidspunktR=2,00] | -3,320198 | 4,341033 | 24,268 | -,765 | ,452 | -12,274425 | 5,634030 |
| [tidspunktR=3,00] | 0^b^ | 0 | . | . | . | . | . |
| trt | 4,549605 | 5,256647 | 22,196 | ,865 | ,396 | -6,346428 | 15,445638 |
| post_trt | 11,492010 | 9,051586 | 23,160 | 1,270 | ,217 | -7,225455 | 30,209475 |
| a. Dependent Variable: SWM3. | | | | | | | |
| b. This parameter is set to zero because it is redundant. | | | | | | | |

| **Estimates of Fixed Effects^a^** | | | | | | | |
| --- | --- | --- | --- | --- | --- | --- | --- |
| Parameter | Estimate | Std. Error | df | t | Sig. | 95% Confidence Interval | |
|  |  |  |  |  |  | Lower Bound | Upper Bound |
| Intercept | 23,452399 | 7,014867 | 28,274 | 3,343 | ,002 | 9,089370 | 37,815429 |
| [tidspunktR=1,00] | -11,087031 | 8,085132 | 26,387 | -1,371 | ,182 | -27,694412 | 5,520351 |
| [tidspunktR=2,00] | -3,256015 | 4,346593 | 23,609 | -,749 | ,461 | -12,234810 | 5,722779 |
| [tidspunktR=3,00] | 0^b^ | 0 | . | . | . | . | . |
| trt | 3,708291 | 6,697192 | 35,965 | ,554 | ,583 | -9,874708 | 17,291291 |
| post_trt | 13,081167 | 10,071955 | 30,481 | 1,299 | ,204 | -7,474907 | 33,637240 |
| [Diagnosis=1] | 10,316641 | 8,197729 | 28,903 | 1,258 | ,218 | -6,452049 | 27,085331 |
| [Diagnosis=2] | 0^b^ | 0 | . | . | . | . | . |
| [Diagnosis=1] * trt | ,895646 | 5,537501 | 43,874 | ,162 | ,872 | -10,265358 | 12,056650 |
| [Diagnosis=2] * trt | 0^b^ | 0 | . | . | . | . | . |
| [Diagnosis=1] * post_trt | -2,598337 | 6,985933 | 30,174 | -,372 | ,713 | -16,862066 | 11,665392 |
| [Diagnosis=2] * post_trt | 0^b^ | 0 | . | . | . | . | . |
| a. Dependent Variable: SWM3. | | | | | | | |
| b. This parameter is set to zero because it is redundant. | | | | | | | |
